# Establishment of a chemoresistant laryngeal cancer cell model to study chemoresistance and chemosensitization responses via transcriptomic analysis and a tumor‐on‐a‐chip platform

**DOI:** 10.1002/btm2.10741

**Published:** 2025-01-22

**Authors:** Christian R. Moya‐Garcia, Meghana Munipalle, Alain Pacis, Nader Sadeghi, Maryam Tabrizian, Nicole Y. K. Li‐Jessen

**Affiliations:** ^1^ Department of Biomedical Engineering, Faculty of Medicine and Health Sciences McGill University Quebec Canada; ^2^ Canadian Centre for Computational Genomics (C3G) McGill University Montreal Quebec Canada; ^3^ Department of Otolaryngology – Head and Neck Surgery McGill University, MUHC (Royal Victoria Hospital) Montreal Quebec Canada; ^4^ Faculty of Dentistry and Oral Health Sciences McGill University Montreal Quebec Canada; ^5^ School of Communication Sciences and Disorders McGill University Montreal Quebec Canada; ^6^ Research Institute of the McGill University Health Centre Montreal Quebec Canada

**Keywords:** chemoresistance, docetaxel, metformin, laryngeal cancer, hypoxia, senescence, transcriptomics, tumor‐on‐a‐chip

## Abstract

Tumor resistance to chemotherapy is a common cause of cancer recurrence in patients with head and neck squamous cell carcinoma. The goal of this study is to establish and characterize a chemoresistant laryngeal cancer cell model and test its potential utility for chemosensitizing therapy. At the genotypic level, RNA sequencing confirmed that the cells acquired putative resistance with upregulated docetaxel‐resistant (DR) genes (e.g., TUBB3, CYP24A1) and signaling pathways (e.g., PI3K/mTOR, autophagy). For phenotypic analysis, DR cells were co‐cultured with laryngeal fibroblasts in a 2‐channel microfluidic chip that mimics a hypoxic tumor core in vivo. A drug sensitivity test with a chemosensitizer, metformin (MTF), was performed on the laryngeal tumor‐on‐a‐chip. Compared to non‐treated controls, MTF‐primed cancer cells exhibit higher sensitivity to docetaxel (DTX), that is, cell death. Collectively, this resistance‐acquired cell model displayed presumed genotypic and phenotypic profiles of chemoresistance providing a viable option for testing new therapeutic strategies for restoring tumor sensitivity to DTX.

**Translational Impact Statement**

Incomplete understanding of chemoresistance in locally advanced mucosal laryngeal carcinoma leads to overtreatment. Here, we develop docetaxel‐resistant laryngeal cancer cells to understand key molecular mechanisms and chemosensitizing strategies. From such resistant‐acquired cells, molecular targets for inhibition are identified regarding upregulated genes and signaling pathways including PI3K/mTOR and autophagy associated with docetaxel resistance. Tumor‐on‐a‐chip cultures emulate the hypoxic laryngeal tumor core for chemosensitizing evaluation. Metformin is used to sensitize chemoresistant cells to docetaxel encapsulated into mucoadhesive chitosomes causing increased cytotoxicity with prospect clinical target of mucin‐overexpressing tumors.

## 1 INTRODUCTION

The global burden of head and neck cancer (HNC) is estimated as $535 billion USD from cases between 2018 and 2030.^1^ HNC encompasses squamous cell carcinoma in the mucosal tissue found in the oral cavity, nasal cavity, pharynx, larynx, and salivary glands.^2^ The 5‐year overall survival rate for patients with locally advanced HNC has been at 50% for the last three decades.^3^, ^4^ About 65% of patients with locally advanced stages of HNC develop metastatic/recurrent cancer with a 15% rate of developing a second primary tumor in the head and neck anatomical site.^5^ Resistance to treatment is a common cause of cancer recurrence and metastasis, leading to treatment failure.^6^ As a sub‐type of HNC with up to a 40% caseload,^7^ laryngeal cancer affects critical functions such as speaking, swallowing, and breathing.^8^ Laryngeal cancer had an annual incidence rate of 177,422 worldwide in 2019.^9^ This incidence rate is expected to increase 43% by 2035 due to attributable risk factors such as smoking and drinking.^10^

In standard treatment of HNC, taxanes, platinum‐based, and 5‐fluorouracil (TPF) are common chemotherapeutic agents, typically used concomitantly with radiotherapy either in a definitive or adjuvant setting.^7^ Docetaxel (DTX) is a taxane drug that was approved by the United States Food and Drug Administration for HNC treatment in 1996.^11^ When administering DTX as a single agent, positive response rates were found as low as 10% in HNC unrelated to human papillomavirus infection.^12^ This low response rate is primarily attributed to tumor resistance to the drugs used in chemotherapy.^2^, ^12^, ^13^, ^14^

Multiple molecular pathways are plausibly associated with HNC progression and therapeutic resistance, such as alterations in drug efflux transporters, disruptions in apoptotic pathways, augmentation of DNA repair mechanisms, and dysregulation of survival signaling pathways.^15^, ^16^ For instance, upregulation of β‐III tubulin,^17^, ^18^, ^19^ cytochrome P450 (CYP),^17^ and adenosine triphosphate (ATP)‐binding cassette (ABC) transporters^17^, ^20^, ^21^ were linked to increased resistance to DTX in HNC. Combination therapy, often involving two or more drugs or treatment modalities, has been explored to circumvent chemoresistance in head and neck oncology.^14^ For instance, PI3K/mTOR pathway is the most aberrantly activated cancer‐associated signaling pathway with more than a 90% prevalence in head and neck malignancy.^22^ Their upregulation is linked to taxane resistance with downstream effects on the regulation of cell survival, metabolism, and proliferation.^17^, ^20^, ^23^, ^24^, ^25^, ^26^

One treatment strategy is targeting the mammalian target of rapamycin (mTOR) pathway to sensitize resistant cancer cells.^22^ Metformin (MTF), which is an antihyperglycemic agent and first‐line treatment for diabetes, has been found to inhibit mTOR pathway in several cancer types.^27^, ^28^, ^29^ When used as an adjuvant treatment to chemotherapy, MTF decreased resistance to taxane therapies in ovarian tumors, with 20% more epithelial cancer cell death compared to taxane alone after a 2‐day inspection.^27^ In gastric cancer, MTF in combination with DTX or 5‐fluorouracil showed more than 80% reduction in gastric cancer colony numbers after a 10‐day clonogenic inspection.^28^ A randomized clinical trial study also suggested a favorable impact of MTF on enhancing the sensitivity of DTX treatment in patients with metastatic prostate cancer.^29^

In HNC, in vitro evidence has become available for possible anti‐tumor effects of MTF^30^ owing to ongoing clinical trials^31^, ^32^ (e.g., phase I,^4^ phase I/II^33^). An in vitro study in oral cancer reported that MTF inhibits cell proliferation and regulates cancer‐associated pathways affecting mTOR and mitochondrial activity.^30^ Also, MTF has shown regulation of resistant mechanisms during curcumin chemotherapy treatment.^34^ In laryngeal cancer, MTF in combination with 5‐fluorouracil was shown to upregulate genes associated with protein metabolism, CYP, endoplasmic reticulum stress, DNA damage, and apoptosis, as well as genes reducing cell proliferation and migration in vitro.^35^ In locally advanced stage III squamous cell carcinoma of the oral cavity, oropharynx, hypopharynx and larynx, adjuvant MTF has shown improved overall survival and progression‐free survival in phase I/II clinical studies.^4^, ^33^

At present, most laryngeal cancer studies rely on animal models.^36^, ^37^ When laryngeal carcinogenesis is studied by subcutaneous injection into the flank^36^ or armpits^37^ of mice, animal models fail to accurately mimic the disease, as they do not target the anatomical site of the laryngeal mucosa. There remains a need for advancing cell culture models for pre‐clinical assessment in laryngeal cancer research. Meanwhile, existing in vitro laryngeal cancer models are limited to characterizing the tumor microenvironment and the tumor response, both sensitivity and resistance, to chemotherapy.^2^, ^38^, ^39^ Advanced culturing models (e.g., dynamic microfluidic systems), together with high‐throughput sequencing technology (e.g., RNA‐sequencing), would provide an effective tool for systematic drug screening and evaluation.^40^, ^41^, ^42^

In this study, we aimed to develop a DTX‐resistant laryngeal cancer cell model that would help advance the understanding of chemoresistance and the evaluation of new drug design. We implemented an escalating intermittent dose protocol to successfully induce DTX resistance in a commercially available laryngeal cancer cell line. RNA sequencing analysis was performed to characterize DTX‐exposed cells' resistance by measuring the upregulation of DTX‐resistant genes and signaling pathways compared to healthy laryngeal epithelial cells and laryngeal cancer cells with no DTX exposure. For functional analysis, DTX‐resistant cells were co‐cultured with vocal fold stromal fibroblasts on a tumor‐on‐a‐chip device, which was then exposed to a DTX‐only monotherapy or combined MTF/DTX sensitization treatment.

## 2 RESULTS

### 2.1 Inducing and genotyping DTX resistance (DR) in laryngeal cancer cells

#### 2.1.1 Stepwise dose‐escalation exposure induced chemoresistance in laryngeal cancer cells

**FIGURE 1:**
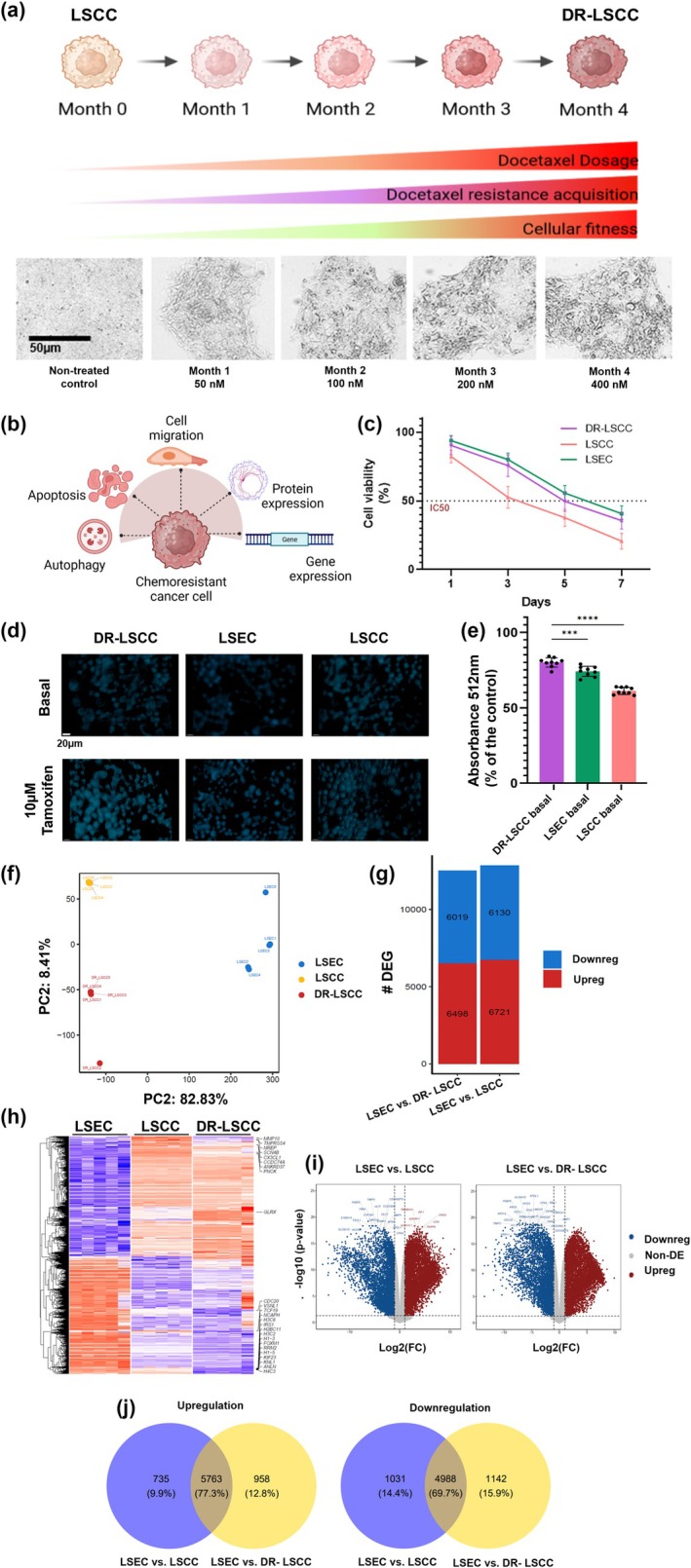
Chemoresistance in laryngeal cancer cells with distinct genotypic profile. (a) Illustration of the protocol for inducing chemoresistance in laryngeal cancer cells. Brightfield images show the progression of chemoresistance and morphological changes in cancer colonies (4× magnification). (b) Chemoresistance can induce autophagy, apoptosis, cell migration, protein expression, gene expression and so on. (c) Drug resistance analysis of DTX cytotoxicity effect on non‐resistant (LSEC and LSCC) and resistant (DR‐LSCC) cells. (d) Autophagy staining of DR‐LSCC, LSCC, and LSEC with positive controls of tamoxifen. Blue = monodansylcadaverine. Scale bar = 20 μm. (e) Normalized autophagy absorbance to positive controls. Ordinary one‐way ANOVA, Bonferroni's multiple comparisons test as post hoc test (*n* = 9, ***p < 0.001, ****p < 0.0001). (f) Principal component analysis (PCA) of the transcriptomic data for each cell group sample (*n* = 5). (g) Total identified differentially expressed genes (DEG) are depicted as heat maps with a Z‐core (−2 to 2). Individual DEGs are represented on the *y*‐axis and the sample regions are along the *x*‐axis. (h) Number of upregulated and downregulated DEG (# DEG) of comparison between cell groups. (i) A volcano plot of LSEC vs. LSCC, and LSEC vs. DR‐LSCC comparisons. (j) Differentially expressed gene Venn diagram of comparison between cell groups. A linear model was used to obtain differentially expressed genes. For all genes and pathways, significance is defined as *p*‐adjusted <0.05. LSEC = laryngeal squamous epithelial cell; LSCC = laryngeal squamous cell carcinoma; DR‐LSCC = docetaxel‐resistant laryngeal squamous cell carcinoma.

DR was induced in laryngeal squamous cell carcinoma (LSCC) by applying a stepwise dose‐escalation exposure protocol, namely, a 3‐day exposure of complete media followed by 3‐day DTX enriched media for 4 months (Figure 1a). Brightfield images showed the evolution of morphological changes in DTX‐exposed cancer colonies over the exposure period, in comparison to parental LSCC (Figure 1a). Chemoresistance was confirmed based on the analyses of apoptosis, autophagy, cell migration, protein and gene expressions (Figure 1b).^17^ The DTX resistance analysis was performed to assess the viability of DR‐LSCC, in comparison with a non‐resistant LSCC group and a healthy laryngeal squamous epithelial cell (LSEC) control group. After a 72‐h of 1 μM DTX exposure, the MTT cell viability assay showed that DR‐LSCC were more resistant to the DTX treatment after a 3‐day inspection. Less cell death was observed in DR‐LSCC than that in LSCCs and LSEC (~36% vs. ~15%; Figure 1c). After confirming an increased resistance on DR‐LSCC, monodansylcadaverine staining was carried out for the detection of autophagy (Figure 1d). Tamoxifen, an autophagy inducer, was used as the positive control.^43^ A significant increase in basal autophagy activity was noted in DR‐LSCC compared to controls (*p* < 0.05), indicating that DR‐LSCC has an increased metabolic activity than LSCC and LSEC (Figure 1e).

#### 2.1.2 DR‐LSCC and LSCC presented overall distinct genotypic profiles compared to LSEC

More than 150 ng/μL of RNA was obtained for all sequencing samples of LSEC, LSCC, and DR‐LSCC (Table S1). Principal component analysis (Figure 1f) was implemented to observe the data in terms of their first dimension (PC1) and second dimension (PC2) of variation between cell groups. PC1 axis was greater than the comparable distances observed along the PC2 axis. PC1 and PC2 accounted for 83% and 8% of variability, respectively, and the three groups of cells were clearly separated from each other. From the 23,922 expressed genes among the three cell groups, differentially expressed genes (DEGs) were identified and depicted as heatmaps to visualize data clustering as entities through a dendrogram and assess the logical coherence of this structure (Figure 1g). Heatmap analysis exhibited distinct genotypic profiles from DR‐LSCC, LSCC, and LSEC of the identified DEG. Healthy LSEC control presented over 12,000 DEG, either upregulated or downregulated, when compared to either LSCC or DR‐LSCC with *p*‐adjusted <0.05 and |log2FC| > 1 (Figure 1h). In total, there were 12,517 DEG between healthy control and cancerous group (LSEC vs. LSCC) and 12,851 DEG between healthy control and resistant cancerous group (LSEC vs. DR‐LSCC).

Volcano plot analysis confirmed the distinctive genotypic profile of both cancerous groups (Figure 1i). Compared to non‐cancerous LSEC, both cancerous groups exhibited statistically significant downregulated (∼40%, displayed as blue dots) and upregulated (∼60%, shown as red dots) genes. To understand the correlation of these data, overlap analysis of up‐ and downregulated DEG from Figure 1g,i was depicted in Venn diagrams (Figure 1j). About 77% (5763/7456) of upregulated DEG and 70% of downregulated DEG (4988/7161) were shared in both cancerous groups compared to non‐cancerous LSEC. Overall, both cancerous groups (LSCC and DR‐LSCC) displayed distinctive genotypic profiles from that of non‐cancerous (LSEC) and subsequent analysis would focus on the comparison of the two cancerous groups.

### 2.2 DR‐LSCC oncogene profiling of metastasis and resistance

**FIGURE 2:**
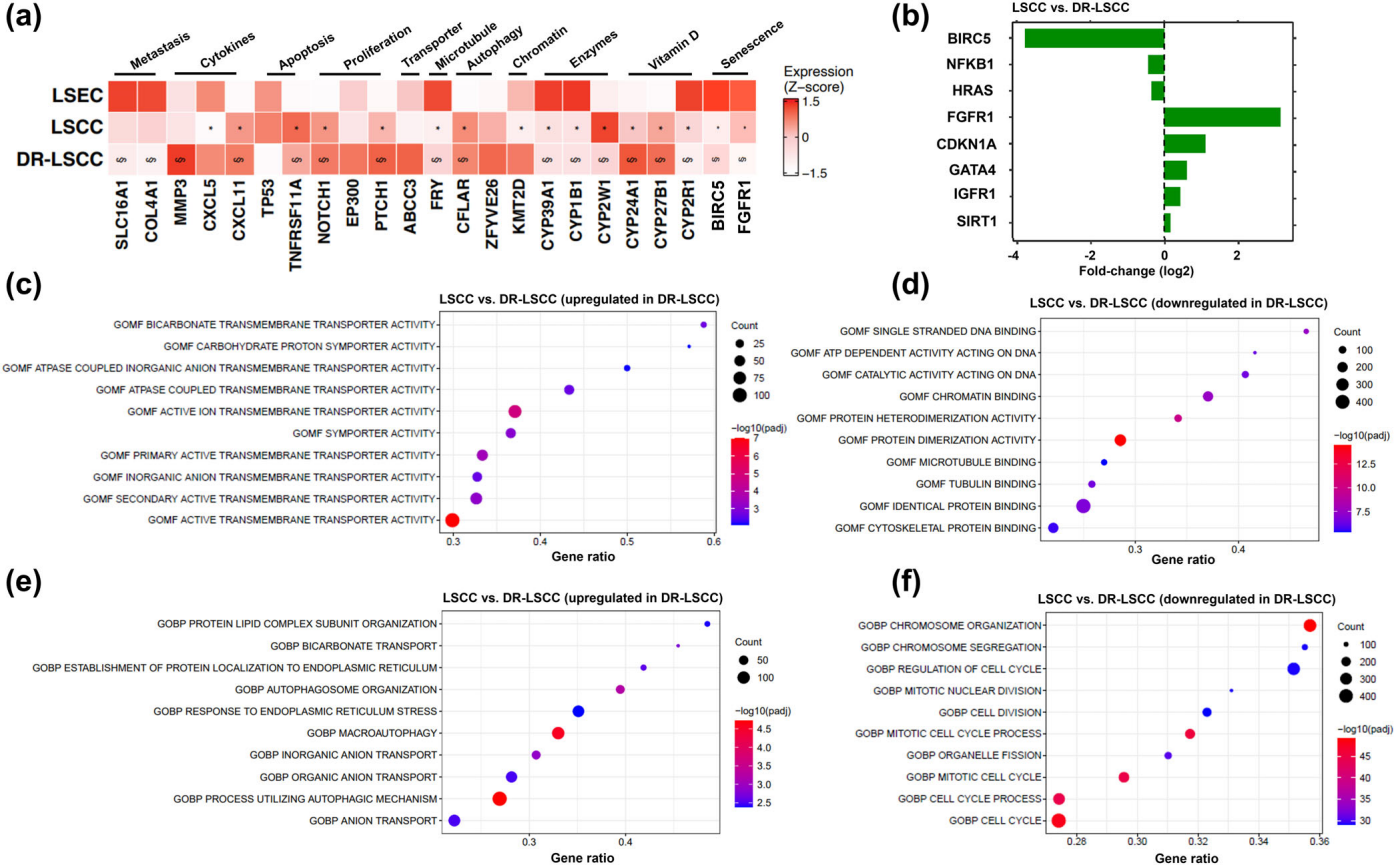
HNC biomarker expression on analyzed laryngeal cells. (a) Heatmap of specific differences on HNC biomarkers, drug metabolism, oncogenes, and tumor suppressors with Z‐score (−1.5 to 1.5), *p < 0.05, § *p* < 0.01 in comparison to LSEC. (b) Relevant chemotherapy‐induced senescence between LSCC and DR‐LSCC with fold‐change (log2). The top 10 gene ontology molecular functions (GOMF) of (c) upregulated and (d) downregulated DEG in DR‐LSCC with ‐log10(*p*‐adj), in comparison to LSCC. Similarly, the top 10 genes biological process (GOBP) of (e) upregulated and (f) downregulated DEG in DR‐LSCC with ‐log10(*p*‐adj). A linear model was used to obtain differentially expressed genes. For all genes and pathways, significance is defined as *p*‐adjusted <0.05.

Based on the analysis of 45 oncogenes known to head and neck squamous cell carcinomas,^17^, ^26^, ^44^, ^45^, ^46^, ^47^, ^48^, ^49^, ^50^, ^51^, ^52^ DR‐LSCC presented a differentially expressed genotypic profile with respect to metastasis cytokine, apoptosis, proliferation, drug transporters, enzymatic, and vitamin D‐related markers compared to LSEC (Figure S1). Of these 45 oncogenes, 21 genes were selected for further investigation herein (Figure 2a) as their expression patterns were distinctive from other related work.^26^, ^45^ Compared to non‐cancerous LSEC, markers of metastasis, namely (wild type) SLC16A1 and COL4A1, were significantly downregulated in DR‐LSCC as anticipated. Such outcome may refer to the constant DTX exposure causing a greater effect on cytokine gene expression as matrix metalloproteinase‐3 (MMP‐3) (*p* < 0.05), CXCL5 (n.s.), and CXCL11 (*p* < 0.05). An explanation may relate to MMP expression being part of the development of metastasis in HNC,^26^, ^45^, ^53^ rather than the lymph node metastatic gene expression of COL4A1 and SLC16A1 as suggested in Jin et al.'s paper.^45^

Further, in comparison to LSEC, apoptosis‐related expression of (wild type) TP53 was downregulated (n.s.) and TNFRSF11A was significantly upregulated (*p* < 0.05) in DR‐LSCC. Genes related to cancer proliferation including NOTCH1 (*p* < 0.05), EP300 (n.s.), and PTCH1 (*p* < 0.05) were all upregulated in DR‐LSCC. Upregulation of the drug transporter ABCC3 (n.s.) and downregulation of microtubule‐binding protein FRY (*p* < 0.05) were noticed in DR‐LSCC. With respect to autophagy‐related genes, DR‐LSCC showed upregulation of CFLAR (*p* < 0.05) and ZFYVE26 (n.s.). A non‐significant upregulation of the chromatin remodeling gene KMT2D was observed in DR‐LSCC.

A group of enzymes known as CYP (Figure 2a, Figure S1) were key players in drug degradation including DTX. The upregulation of CYP enzymes is associated with chemoresistance.^17^, ^54^, ^55^, ^56^ In this study, DR‐LSCC showed significantly lower CYP39A1, CYP1B1 and CYP2W1 expression (Figure 2a) denoting potential decreased degradation capabilities toward DTX compared to LSEC. Although this result was not expected, CYP enzyme downregulation was found in disease states with reduced expression of nitric oxide (NOS)‐associated pathways,^57^ of which were also evidenced by our NOS1 results (Figure S2). Furthermore, CYP2W1 may play a role in the DTX‐resistant phenotype considering oncoPrint data analysis showing low CYP2W1 gene expression in head and neck squamous cell carcinoma (stages I–IV)^56^ as exhibited by LSEC and DR‐LSCC. Vitamin D‐mediated biological mechanisms may regulate cancer cell metabolic and survival activities. Significant upregulation of vitamin‐D‐related genes CYP24A1 and CYP27B1 and downregulation of CYP2R1 (all *p* < 0.05) was observed in DR‐LSCC. This result is expected given the adverse prognostic indicators of impaired vitamin D for HNC progression.^55^, ^58^, ^59^ Nonetheless, increased CYP24A1 with reduced (wild type) TP53 expression is a key enzymatic process mediated by the vitamin D pathway,^55^ which was exhibited by DR‐LSCC in this study.

Continuous DTX exposure was expected to drive DR‐LSCC to a senescent state.^60^ Chemotherapy‐induced senescence contributes to drug resistance and cancer aggressiveness when cancer cells survive successive chemotherapy treatments.^61^ Compared to LSEC, both cancerous groups (DR‐LSCC and LSCC) exhibited significant downregulation of chemotherapy‐induced senescence‐related genes BIRC5^60^ and FGFR1^62^ (all *p* < 0.05), (Figure 2a). Interestingly, between the two cancerous groups, DR‐LSCC showed significant downregulation of BIRC5 but upregulation of FGFR1 (Figure 2b). Specifically, DR‐LSCC showed a ~3.8‐log2 fold downregulation of BIRC5 that encodes survivin—a family member of apoptosis inhibitor and spindle assembly checkpoint.^60^ Moreover, significant <0.4‐log2 fold downregulation of transcription factor NFKB1 and proto‐oncogene HRAS was noted in DR‐LSCC, potentially related to senescence‐associated secretory phenotype and oncogene‐induced senescence.^62^ In addition to FGFR1 (~3.1 log2 fold‐change), DR‐LSCC exhibited significant upregulation of CDKN1A (~1.1 log2 fold‐change), GATA4 (~0.6 log2 fold‐change), IGF1R (~0.4 log2 fold‐change), and SIRT1 (~0.2 log2 fold‐change), attributed to cellular senescence upregulating PI3K/mTOR and inactivating P53 respective pathways.^62^

#### 2.2.1 Gene ontology (GO) analysis revealed unique molecular pathways between LSCC and DR‐LSCC

To gain a better understanding of the functional differences between the two cancerous groups, we performed differential expression analysis of DR‐LSCC relative to LSCC (Figures S2 and S3). We next applied gene set enrichment analysis (GSEA) using GO annotations. Based on GO molecular functions (GOMFs), DR‐LSCC showed significant upregulation on transmembrane transporter and symporter activities than LSCC (Figure 2b) while molecular functions including DNA activity, protein dimerization, and cytoskeleton binding are downregulated (Figure 2c). For GO biological processes (GOBPs), the most upregulated gene sets in DR‐LSCC compared to LSCC were involved in autophagic mechanism, anion transport, and endoplasmic reticulum responses (Figure 2d), while cell cycle is the top downregulated process (Figure 2e). These biological processes are known to implicate in the metabolism and differentiation of laryngeal cancer development and progression,^63^ confirming the acquired chemoresistance of DR‐LSCC at the gene transcription level. The ability of cancer cells to alter their metabolism has been associated with their resistance to anti‐cancer drugs. In particular, resistant cells were able to adapt to oxidative stress and balance their internal redox levels by boosting the production of glutathione.^64^ Such metabolic adaptation can be attributed to the mutation or deregulation of some genes that control microtubule stabilization, cancer senescence, hypoxia and antioxidant capacity, and adaptive mitochondrial reprogramming as described next.

### 2.3 Chemoresistance‐related genes and pathways: in vitro verification

#### 2.3.1 DR‐LSCC expressed chemoresistant‐associated genotypes

**FIGURE 3:**
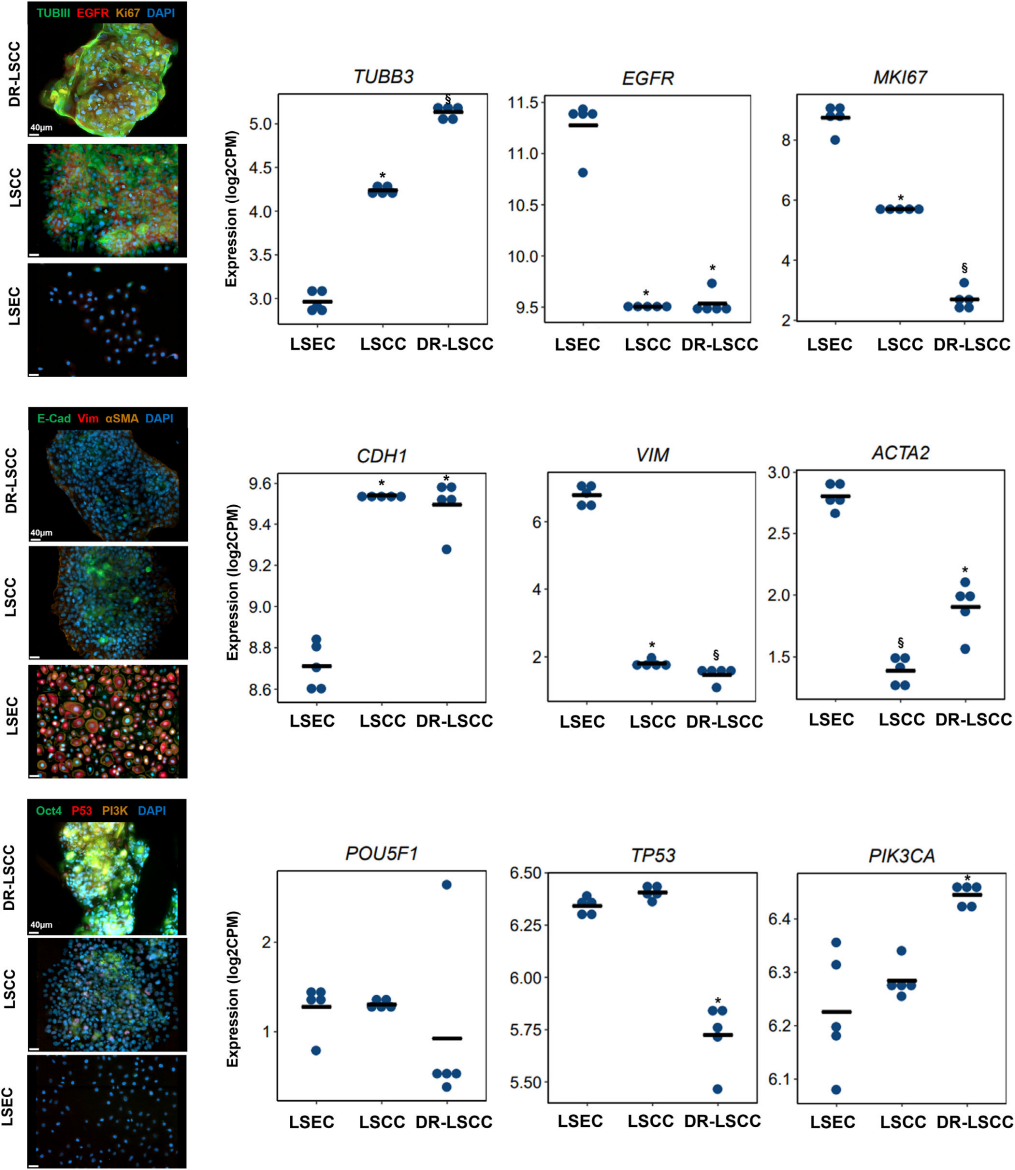
Immunostaining and corresponding transcriptomic data of chemoresistance markers. Immunofluorescence of βIII‐tubulin (TUBIII), EGFR, Ki‐67, E‐cadherin, vimentin, α‐smooth muscle actin (α‐SMA), Oct‐4, P53 and PI3K with their specific gene expression. Scale bar = 40 μm. Gene set enrichment analysis (GSEA) based on a pre‐ranked gene list by t‐statistic (*n* = 5, *p* < 0.05, § *p* < 0.01 in comparison to LSEC). A linear model was used to obtain differentially expressed genes.

Overall, compared to those of LSEC and LSCC, DR‐LSCC showed upregulated expression of microtubule stabilization genes like ALK gene, metabolic gene GSK3B, and a slight increase of hypoxia gene HIF1A in the KEGG hsa05200 cancer pathway (Figures S2 and S3). The result is in concordance with a presumed highly metabolic and hypoxic phenotype of resistant cells reported in literature.^17^ A set of chemoresistance‐related markers,^15^, ^17^, ^20^, ^22^, ^23^, ^65^, ^66^, ^67^, ^68^, ^69^ namely βIII‐tubulin (TUBIII), EGFR, Ki‐67, epithelial cadherin (E‐cad), vimentin, α‐smooth muscle actin (α‐SMA), Oct‐4, P53, and PIK3CA were further profiled and verified in this study. (Figure 3, Figure S4).

For microtubule marker of TUBIII, both transcriptomic and staining results showed increased expression in DR‐LSCC, attributable to DTX microtubule‐binding effect.^70^ For HNC oncogene of EGFR, immunostaining showed strong expression on the cell surface of DR‐LSCC and LSCC but in opposite expression from the transcriptomic results. It is possible that continuous exposure to DTX may have a plateau effect on EGFR expression at the gene level. These results were unexpected since EGFR overexpression is often associated with taxane drug resistance.^16^ For the proliferation marker of Ki‐67, both cancerous groups showed strong stains but their encoding gene MKI67 was significantly downregulated compared to LSEC (*p* < 0.05). Although low levels of Ki‐67 are associated with cancer senescence,^71^ DR‐LSCC exhibited proliferative activity via Ki‐67 immunofluorescent inspection contradicting the MKI67 gene results.

For a cell adhesion marker of CDH1 gene and its encoding E‐cad, both cancerous groups showed significantly stronger expression than LSEC (*p* < 0.05). For a mesenchymal marker of vimentin, both gene and staining data showed a low expression of this focal adhesion marker in DR‐LSCC and LSCC. Another mesenchymal marker of ACTA2 gene and its α‐smooth muscle actin, both cancerous groups also showed downregulation compared to LSEC (*p* < 0.05), whereas DR‐LSCC had significantly higher ACTA2 expression compared to LSCC. Chemotherapy‐induced cancer senescence reprograms metabolic activity resulting in promotion of the epithelial‐mesenchymal transition,^72^ which has been associated with oral squamous cell carcinoma promotion and invasiveness.^73^

For stemness markers of POU5F1 gene and its Oct‐4, all cell types had comparable expressions. With respect to the tumor suppressor marker of TP53 gene and its P53, DR‐LSCC showed significantly lower expression than the other two cell groups (*p* < 0.05). Such results were anticipated considering that reduced expression of TP53 manifested the process of cancer senescence.^71^ For a metabolic mTOR‐related marker of PIK3CA encoding gene and its P13K, DR‐LSCC showed higher expression than LSEC. Collectively, these results suggested the genotypic senescent state of DR‐LSCC could be linked to a higher possibility of survival from drug challenges.

#### 2.3.2 DR‐LSCC showed upregulated mTOR‐signaling pathways and functional processes

**FIGURE 4:**
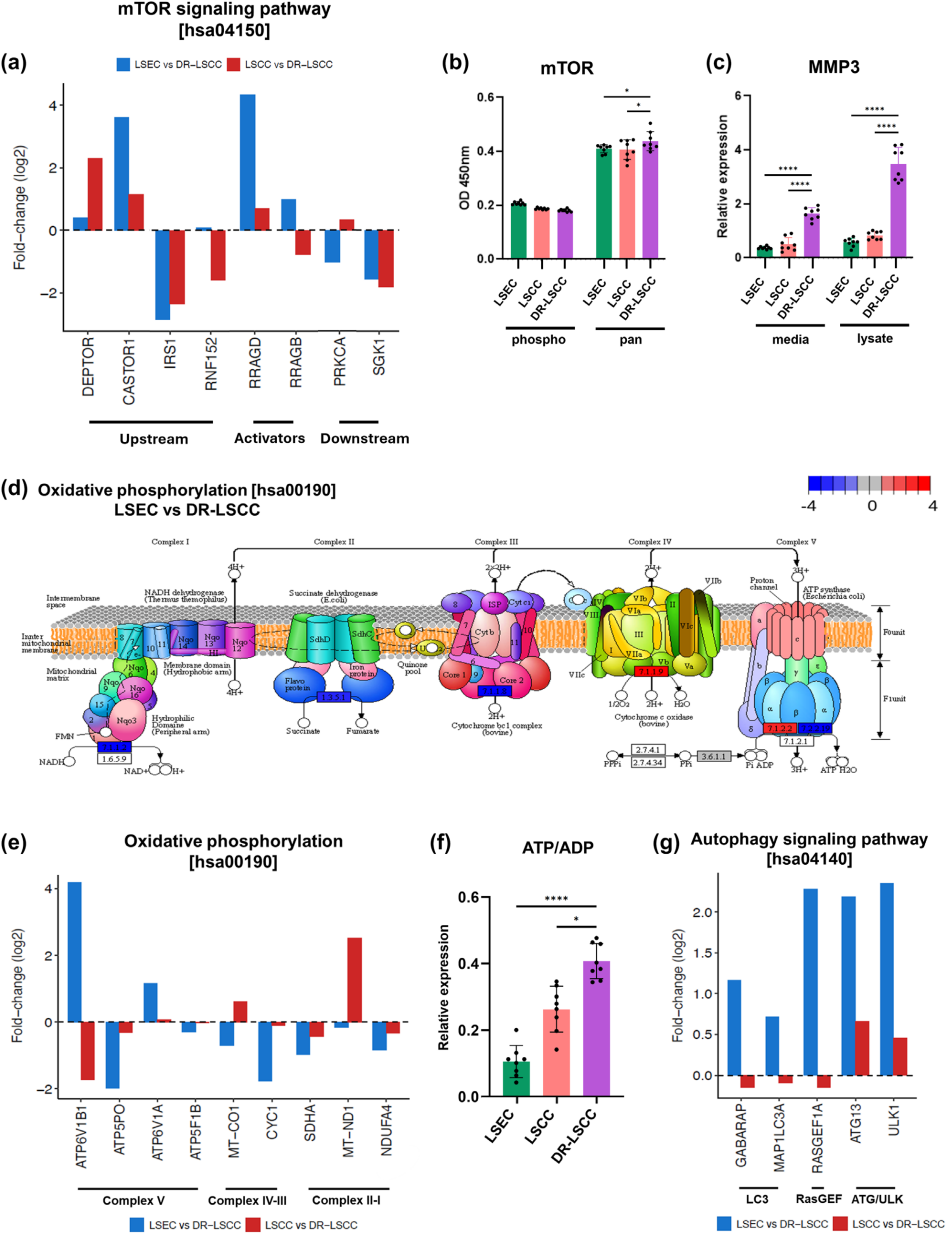
mTOR, oxidative phosphorylation, and autophagy signaling pathways. (a) Genes of interest from mTOR pathway [hsa04150]. Blue = LSEC vs. DR‐LSCC; Red = LSCC vs. DR‐LSCC. (b, c) ELISA data of mTOR and MMP3 expressions of the cell groups LSEC, LSCC and DR‐LSCC. Two‐way ANOVA, Bonferroni's multiple comparisons test as post hoc test (*n* = 8, *p < 0.05, ****p < 0.0001). (d) KEGG oxidative phosphorylation pathway [hsa00190] on non‐resistant and resistant cells (LSEC vs. DR‐LSCC) with color of fold‐change (−4 to 4). Red = upregulation; Blue = downregulation. Genes of interest from KEGG oxidative phosphorylation pathway [hsa00190]. Blue = LSEC vs. DR‐LSCC; Red = LSCC vs. DR‐LSCC (f) Luminescent data on ATP/ADP ratio of LSEC, LSCC, and DR‐LSCC. Ordinary one‐way ANOVA, Bonferroni's multiple comparisons test as post hoc test (*n* = 8, *p < 0.05, ****p < 0.0001). (g) Genes of interest from KEGG autophagy pathway [hsa04140]. Blue = LSEC vs. DR‐LSCC; Red = LSCC vs. DR‐LSCC. A linear model was used to obtain differentially expressed genes. For all genes and pathways, significance is defined as *p*‐adjusted <0.05.

Upregulation of the mTOR complex^17^, ^74^, ^75^, ^76^, ^77^, ^78^ is associated with resistance to taxane drugs.^17^ Compared to LSEC and LSCC, DR‐LSCC showed respective 0.5‐log2 fold and 2.2‐log2 fold increases in the upstream DEPTOR based on the KEGG Pathway analysis [hsa04150 mTOR signaling] (Figure 4a, Figure S5a). CASTOR1 was also upregulated in DR‐LSCC, compared to both LSEC (~3.8 log2 fold‐change) and compared to LSCC (~1 log2 fold‐change). In contrast, DR‐LSCC showed downregulation of IRS1 in comparison to both controls (>2.2 log2 fold‐change). Meanwhile, RNF152 exhibited a ~0.2‐log2 fold increase and ~1.5‐log2 fold decrease in DR‐LSCC compared to LSEC and LSCC respectively.

For mTOR transcriptional activators, DR‐LSCC showed upregulation for RRAGD compared to LSEC (~4 log2 fold‐change) and LSCC (~0.7 log2 fold‐change). For RRAGB, DR‐LSCC showed about a 1‐fold increase and 0.8‐log2 fold decrease compared to LSEC and LSCC respectively. PRKCA is an mTOR downstream target, which showed a ~1‐log2 fold decrease and ~0.5‐log2 fold increase in DR‐LSCC compared to LSEC and LSCC respectively. SGK1 showed a decreased expression (~1.5 log2 fold‐change) in DR‐LSCC in comparison to the other two cell groups. SKG1 dysregulation is tumor‐specific, being upregulated in oral squamous cell carcinomas and downregulated in colorectal cancer.^79^

Intracellular mTOR proteins were quantified to verify the mTOR ontology results using enzyme‐linked immunosorbent assays (ELISAs). Compared to LSCC and LSEC, DR‐LSCC showed significant protein levels of pan‐mTOR but not phosphorylated ones, indicating higher metabolic activity and acquired chemoresistance (Figure 4b). One downstream effect of mTOR signaling is to regulate cytokine production in cancer cells.^45^ For instance, MMP‐3 cytokine is an HNC oncogene that is involved in tumor remodeling.^45^ DR‐LSCC showed significant increases of MMP‐3 in both secreted and intracellular protein levels from media and lysate samples respectively, compared to LSCC and LSEC (*p* < 0.05, Figure 4c). Together, these results may relate to the DR‐LSCC's upregulation of PI3K/mTOR‐related (i.e., PIK3CA: 0.2‐log2 fold increase; PRKCA: 0.3‐log2 fold increase) as well as cytokine‐related (i.e., STAT1‐4 complex: varied between 1.7 and 0.7‐log2 fold increase; NOS1: 2.7‐log2 fold increase) pathways (Figure 4, Figures S2 and S3).

#### 2.3.3 DR‐LSCC showed upregulated mitochondrial and autophagy pathways

Pathway analysis of mitochondrial activity as oxidative phosphorylation [hsa00190] was evaluated between resistant and non‐resistant cells in terms of ATPase, ATP synthase, hydrogenase, oxidase, and reductase expressions (Figure 4d,e). For Complex V ATPase ATP6V1B1, DR‐LSCC showed about a 4‐fold increase and a 2‐fold decrease compared to LSEC and LSCC respectively. For F‐type ATPase ATP5PO, DR‐LSCC showed downregulation than those of LSEC (~2 log2 fold‐change) and LSCC (~0.3 log2 fold‐change) controls. Regarding H+ ATPase ATP6V1A, DR‐LSCC exhibited upregulation compared to LSEC (~1.2 log2 fold‐change) and LSCC (~0.2 log2 fold‐change). For membrane ATP synthase ATP5F1B, DR‐LSCC showed <0.5‐log2 fold downregulation compared to the other two groups. With regards to the mitochondria's Complex IV and III, these cytochrome c‐related genes may play a role in the modification of energetic metabolism in DR‐LSCC. For the oxidase MT‐CO1, DR‐LSCC showed about ~0.6‐log2 fold decrease and ~ 0.5‐log2 fold increase compared to LSEC and LSCC respectively. For reductase CYC1, DR‐LSCC exhibited about 2‐log2 fold and 0.3‐log2 fold decreases than those of LSEC and LSCC.

Complex II‐related SDHA, Complex I‐related MT‐ND1 and NDUFA4 are the genes that may mediate dehydrogenase and oxidoreductases in chemoresistance^31^, ^80^ (Figure 4e). For succinate dehydrogenase SDHA, DR‐LSCC showed downregulation when compared to LSEC (~1 log2 fold‐change) and LSCC (~0.5 log2 fold‐change). For Complex I oxidoreductase MT‐ND1, DR‐LSCC showed about a 0.4‐log2 fold decrease and about ~2.5‐log2 fold increase compared to LSEC and LSCC respectively. The upregulation of MT‐ND1 may affect encoding‐enzyme groups of mitochondria's Complex I for cancer cell respiratory capacity. For oxidoreductase NDUFA4, DR‐LSCC showed downregulation of this oxidative‐stress‐related gene, when compared to LSEC (~0.9 log2 fold‐change) and LSCC (~0.4 log2 fold‐change) controls. The metabolic activity of DR‐LSCC was confirmed at the intracellular protein level. By implementing a bioluminescent assay on cell lysates, DR‐LSCC showed a significant increase in relative expression of baseline ATP/ADP ratio (*p* < 0.05, 1‐fold), compared to those of LSCC and LSEC (Figure 4f). This intracellular protein data confirmed the likely association of increases in metabolic genes (e.g., PI3KCA, GSK3B, CYP24A1, etc.) and ATP/ADP energy activity (ATP6V1A) in DR‐LSCC.

Besides metabolic reprogramming, autophagy is also associated with taxane chemoresistance.^15^, ^17^, ^20^, ^81^, ^82^ Based on the pathway analysis of autophagy [hsa04140] (Figure 4g, Figure S5b), when compared to healthy cells (i.e., LSEC), the five autophagy genes (GARABAP, MAP1LC3A, RASGEF1A, ATG13, and ULK1) were all upregulated in DR‐LSCC. However, when compared to non‐resistant cancer cells (i.e., LSCC), only two of them, namely, ATG3, and ULK1 were upregulated, whereas the other three were downregulated in DR‐LSCC. Resistance often involves aberrations in apoptosis regulation. Downregulation of pro‐apoptotic elements (e.g. MDM2), and upregulation of anti‐apoptotic proteins (e.g., BCL2) may confer a survival advantage to cancer cells^67^ in response to DTX (Figures S2 and S3).

#### 2.3.4 An mTOR‐inhibitor exposure study further confirmed the phenotype of DR‐LSCC

MTF is a rapamycin agent that inhibits mTOR that has demonstrated the ability to enhance the sensitivity of tumor cells to chemotherapy drugs.^27^, ^28^, ^29^, ^83^ MTF also inhibits the mitochondrial oxidative phosphorylation by targeting the Complex I NADH dehydrogenase,^31^, ^80^ such as upregulated MT‐ND1 (Figure 4e). MTF was thus chosen to validate the phenotype of DR‐LSCC via a cell sensitization analysis.

**FIGURE 5:**
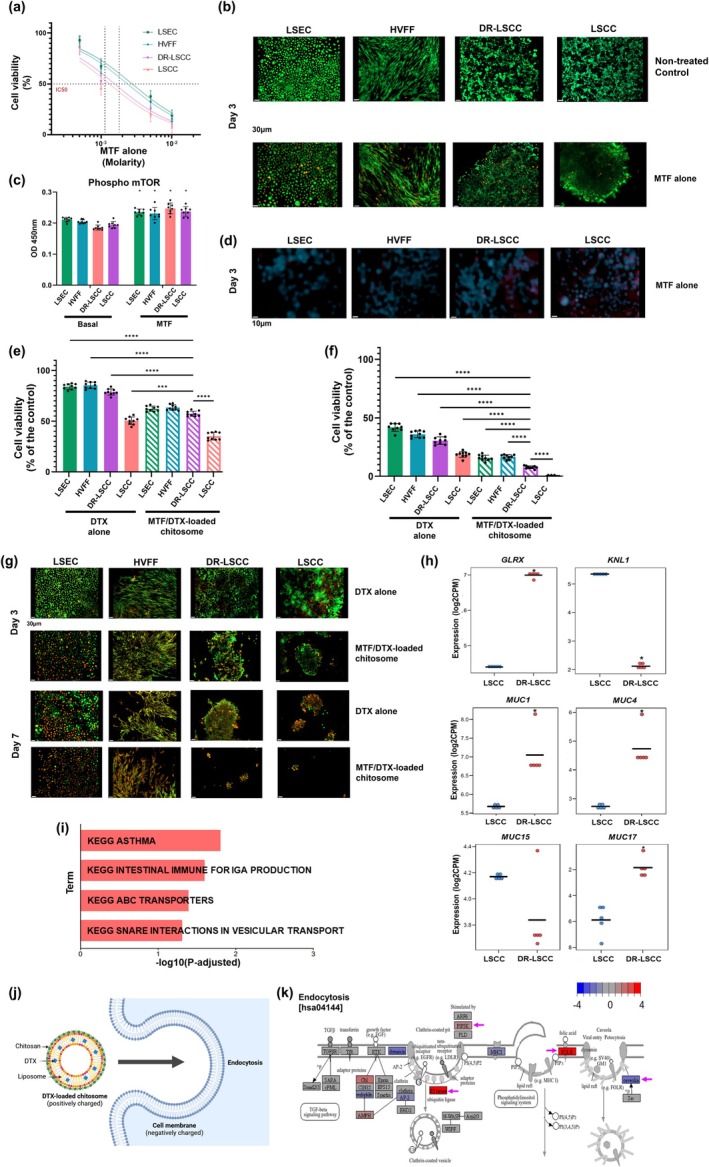
Effects of MTF alone and its combination with DTX on non‐resistant and resistant cells. (a) Dose response curve of the MTF dosage on non‐resistant cells and DR‐LSCC. Relative‐Absolute IC50 threshold expressed as vertical dotted lines. (b) Cytotoxic evaluation of MTF dosage via LIVE/DEAD staining. Green = live cells; Orange = dead cells. Scale bar = 30 μm. (c) Phospho‐mTOR levels after 1 mM metformin. Two‐way ANOVA, Bonferroni's multiple comparisons test as post hoc test (*n* = 9, *p < 0.05 compared to basal controls). (d) Autophagy activity after 1 mM metformin. Blue = autophagy activity; Red = dead cells. Scale bar = 10 μm. MTT analysis on cell viability after 3‐Day (e) and 3‐Day (f) treatment exposures. Two‐way ANOVA, Bonferroni's multiple comparisons test as post hoc test (*n* = 9, ***p < 0.001, ****p < 0.0001 compared to DR‐LSCC with combination therapy). (g) Cytotoxic evaluation of DTX alone, and MTF/DTX‐loaded chitosomes via LIVE/DEAD staining. Green = live cells; Orange = dead cells. Scale bar = 30 μm. (h) Genes associated with antioxidant, microtubule binding, and mucus glycosylation activities. Gene set enrichment analysis (GSEA) based on a pre‐ranked gene list by t‐statistic (*n* = 5, *p < 0.05). (i) KEGG analysis of DR‐LSCC upregulated genes compared to LSCC. (j) Schematic representation of DTX‐loaded chitosome internalization by cells. (k) Representative KEGG endocytosis pathway [hsa05200] LSCC vs. DR‐LSCC, with genes of interest PIP5K1A, E3 ligase/UBR3, FOLR1, CAV1/caveolin (pointed at with magenta arrows) with color of fold‐change (−4 to 4). Red = upregulation; Blue = downregulation. A linear model was used to obtain differentially expressed genes. For all genes and pathways, significance is defined as *p*‐adjusted <0.05.

DR‐LSCC, LSEC, LSCC and stromal human vocal fold fibroblasts (HVFF) were exposed to 500 mM, 1 mM, 5 mM and 10 mM MTF for 3 days. MTF cytotoxic activity was evaluated via mitochondrial‐related MTT assay (Figure 5a) and LIVE/DEAD staining (Figure 5b). Results showed a decreasing trend in cell viability after exposing the cell to the higher concentration of MTF after the 3‐day inspection. Based on the results above, 1 mM MTF treatment was selected for subsequent experiments due to its therapeutic effect below IC50 for the cancerous groups, but above IC50 threshold for non‐cancerous LSEC and stromal HVFF.

ELISA data showed that 1 mM of MTF treatment significantly (*p* < 0.05) enhanced the presence of phosphorylated mTOR in the four cell groups, suggesting an inhibitory effect on mTOR synthesis (Figure 5c). Autophagy evaluation using monodansylcadaverine and propidium iodide stain showed a noticeable increase of autophagy in MTF‐treated DR‐LSCC and compared to HVFF and LSEC (Figure 5d).

Upon cells were sensitized with MTF chemosensitizer followed by DTX‐loaded mucoadhesive chitosomes (Figure 5e–g, Figure S6), MTF‐primed DR‐LSCC showed more cell death (<25% viable cells, *p* < 0.05) at Day 3 and Day 7, compared to DTX alone groups (Figure 5e–g). Similar patterns were also observed in LSCC and non‐cancerous cells, indicative of restoring drug sensitivity from MTF.

#### 2.3.5 DR‐LSCC showed upregulated antioxidant, vesicle transport and mucin‐related genes that may facilitate treatment and chitosome internalization

Compared to LSCC, DR‐LSCC showed an upregulated antioxidant‐associated GLRX^84^ gene, downregulated microtubule‐related KNL1 gene,^85^ as well as upregulated biological processes related to ABC transporters and SNARE interactions in vesicular transport (Figure 5h,i). These genes and biological processes were related to drug sensitivity and retention of the encapsulated DTX in chitosomes.

Further, in comparison with LSCC, mucosal glycosylation^86^ MUC1, MUC4 and MUC17 along with MUCL1, MUC2, and MUC5AC were significantly overexpressed (*p* < 0.05, >1 log2 fold increase), whereas MUC15 was the only mucin‐related gene downregulated (*p* > 0.05, 0.2 log2 fold decrease) in DR‐LSCC (Figure 5h, Figure S7). Upregulation of membrane genes associated with endocytosis, that is, PIP5K1A, E3 ligase/UBR3, FOLR1, and mucosal barrier formation was also observed in DR‐LSCC, which are likely favorable for the mucoadhesive chitosome internalization by DR‐LSCC (Figure 5j,k). The observed enhanced drug sensitivity may relate to clathrin‐dependent endocytic internalization, being CAV1/caveolin downregulated (Figure 5k), of quasi‐targeted mucoadhesive chitosomes (Figure 5j,k) as potentially explained by the transcriptomic profile.

Further, our DR‐LSCC cell model showed the preservation of resistant phenotype after one freeze–thaw cycle. DTX alone and combination therapy were exposed to both DR‐LSCC and thawed DTX‐resistant cells (tDR‐LSCC). LIVE/DEAD staining (Figure S8a), MTT readouts (Figure S8b) and clonogenic analyses (Figure S8c,d) showed that tDR‐LSCC displayed a similar percentage of cell death and colonies as DR‐LSCC after Day 3 and Day 7 of both DTX alone and MTF/DTX treatments. Specifically, the combination therapy exhibited 15% more cell death in DR‐LSCC and tDR‐LSCC after 3‐day and more than 20% of cell death was observed after 7 days, compared to DTX alone (*p* < 0.0001; Figure S8b,d). Autophagy activity and propidium iodide staining showed both DR‐LSCC and tDR‐LSCC had increased autophagy with decreased cell death compared to LSCC after DTX alone and DTX‐loaded chitosome treatments after 7 days (Figure S8e).

### 2.4 DR‐LSCC sensitization treatment on laryngeal tumor‐on‐a‐chip platform

Hypoxic tumor core, which is an important resistant‐related tumor microenvironment feature, was not represented in the prior 2D monolayer study. To emulate the hypoxic laryngeal tumor core,^2^ we cultured DR‐LSCC with HVFF under a diffusion/hypoxic gradient in a microfluidic platform. The chip cultures were then subjected to a new set of chemosensitizing studies for 5 days.

#### 2.4.1 Tumor‐on‐a‐chip co‐culture cells presented a hypoxic marker

**FIGURE 6:**
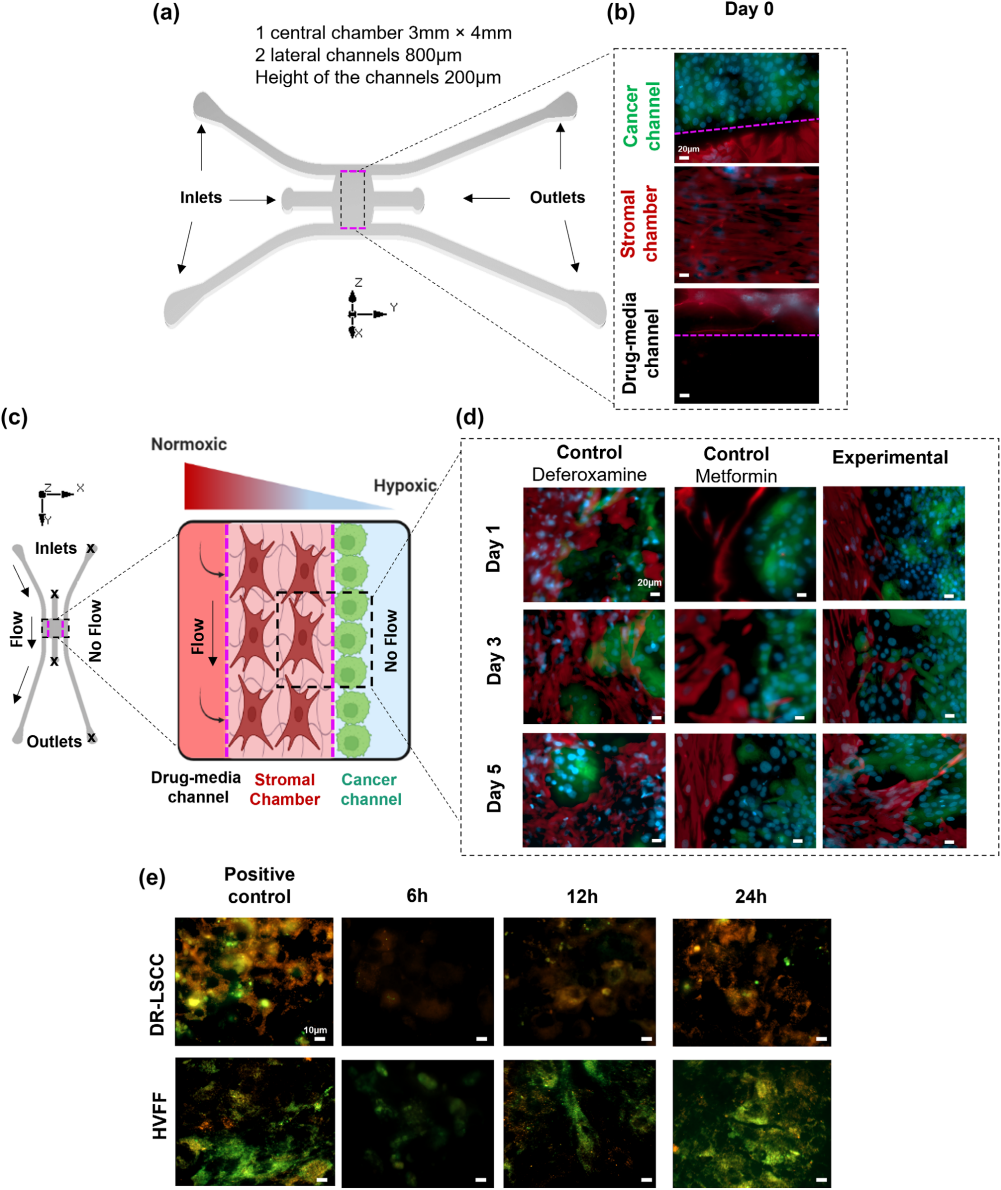
Hypoxia and migration analyses within the microfluidic device. (a) CAD design of the BEOnChip Gradient device. DR‐LSCC were seeded on mucin‐coated cancer channels, whereas HVFF embedded in a collagen I gel were placed in a Stromal chamber. (b) Phenotypic markers of the co‐culture at Day 0. DR‐LSCC (green/TUBIII^+^, red/Vimentin^−^) and HVFF (green/TUBIII^−^, red/Vimentin^+^). Magenta line = Collagen I gel limit. (c) Schematic representation of the co‐culture setup within the microfluidic device mimicking the hypoxic tumor core by blocking inlets/outlets (represented as ×) after cell seeding to create an oxygen/nutrient gradient flow. (d) Migration analysis of the stromal cells (red, vimentin) toward the cancer channel (green, TUBIII), scale bar = 20 μm. Controls of Deferoxamine as hypoxia inducer and MTF as proliferation inhibitor (e) Hypoxia and oxidative stress analyses on DR‐LSCC and HVFF at 24 h (Day 0). Orange = hypoxia; Green = oxidative stress. Positive controls: Deferoxamine as hypoxia inducer and Pyocyanin as oxidative stress inducer.

The DR‐LSCC/HVFF co‐culture was performed on a microfluidic device with two microchannels and one central chamber (Figure 6a). On Day 0, results of phenotypic markers confirmed the co‐culture of DR‐LSCC (green/TUBIII^+^, red/Vimentin^−^) and HVFF (green/TUBIII^−^, red/Vimentin^+^) in their shared distinct compartments (Figure 6b). The diffusion/hypoxic gradient was set up by blocking the inlets/outlets of the stromal chamber and cancer channel after cell seeding (Figure 6c). By doing that, the oxygen/nutrients diffused from the drug‐media channel toward the co‐culture setup. After 5 days of co‐culture (Figure 6d), HVFF were observed to migrate toward the cancer channel when a hypoxia inducer (deferoxamine) was introduced as a positive control. In the negative control group, MTF was used to inhibit HVFF migration owing to its antiproliferative effect on stromal cells known in the literature.^87^ In our experimental group, HVFF were observed to migrate toward DR‐LSCC, similar to the positive control groups. Also, a slight DR‐LSCC growth into the stromal channel was observed, along with the HVFF migrating into the cancer channel (Figure 6d). This could be related to a slight increase in nitroreductase activity of hypoxic DR‐LSCC and more oxidative stress on HVFF (Figure 6e), owing to the hypoxia gradient in the microfluidic device.

#### 2.4.2 DR‐LSCC/HVFF co‐culture showed increased sensitivity to combination therapy

**FIGURE 7:**
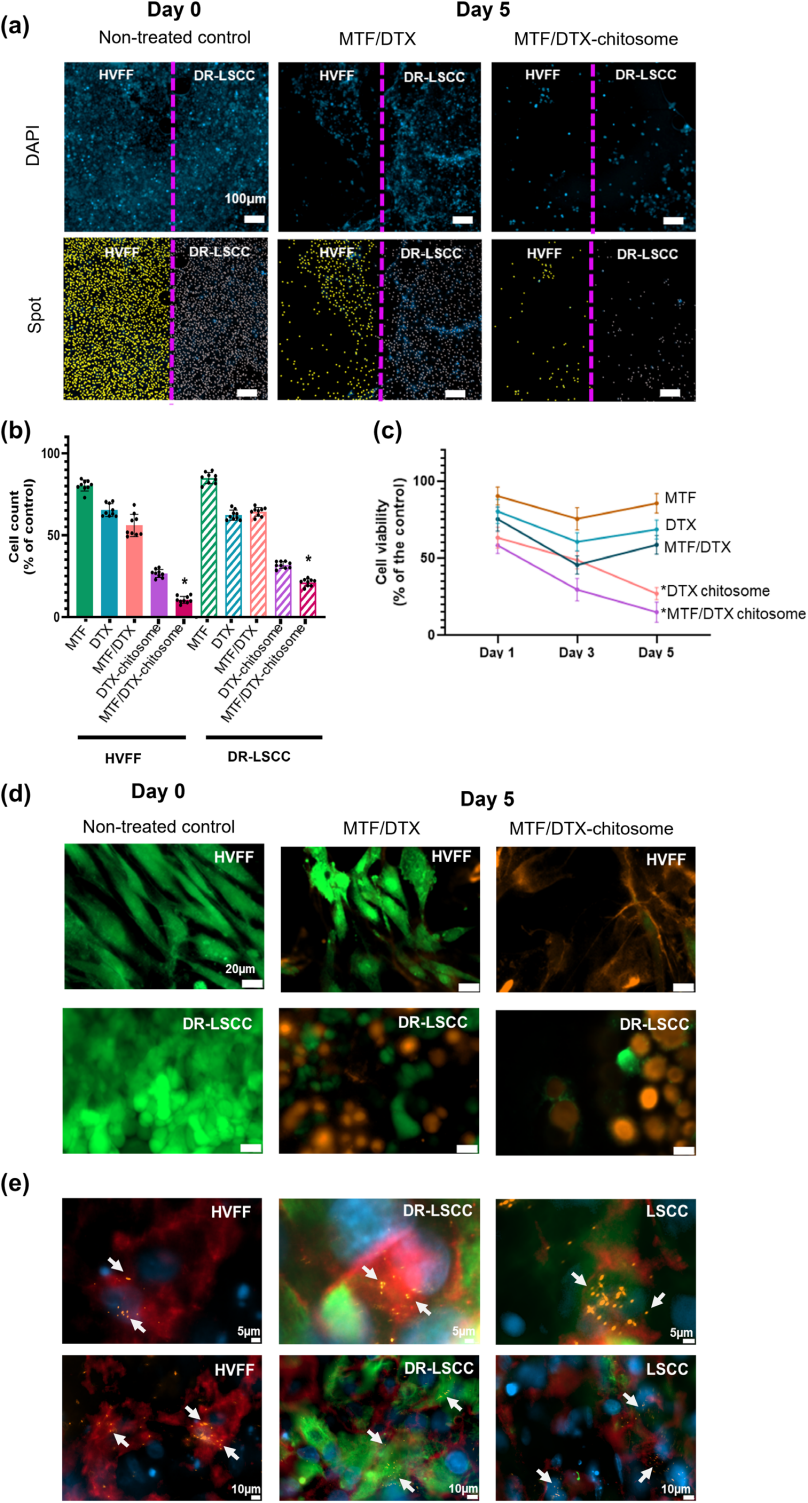
MTF/DTX combination therapy tested on the tumor‐on‐a‐chip. (a) Cytotoxic effect via DAPI inspection, and Spot detection algorithm at Day 5. Yellow spots = HVFF; Gray spots = DR‐LSCC; Magenta line = collagen gel limit. Scale bar = 100 μm. (b) Cell count using Spot detection algorithm on DAPI images at 5‐day inspection. Two‐way ANOVA, Bonferroni's multiple comparisons test (*n* = 9, *p < 0.05 compared to respective cell groups with combination therapy). (c) LDH co‐culture supernatant analysis. One‐way ANOVA and Bonferroni's multiple comparisons as post hoc test (*n* = 9, *p < 0.05). (d) LIVE/DEAD images on Day 5. Green = live cells; Orange = dead cells. Scale bar = 20 μm. (e) Fluorescent chitosome uptake. Immunostaining of HVFF (red/EGFR^+^, green/CK5^−^) and cancer (red/EGFR^+^, green/CK5^+^) cells showed the internalization of the orange‐fluorescent chitosomes (pointed at with white arrows) with DAPI as counterstaining after 6 h inspection. Cancer‐associated fibroblast behavior characterized by EGFR expression could have been activated by DR‐LSCC/HVFF crosstalk (cytokine pathway).^88^, ^89^ Scale bars = 2 μm (top images), 10 μm (below images).

Chip cultures with DR‐LSCC and HVFF were exposed to a combined MTF/DTX treatment for up to 5 days. The administration of the drugs was a 6‐h MTF priming and then a 12‐min perfusion of DTX or DTX‐loaded chitosomes, followed by 18 hrs of MTF perfusion. Overall, cell count analysis based on DAPI showed that HVFF and DR‐LSCC decreased by number after treatment (Figure 7a,b, Figure S9, Video S1). The LDH results further showed that the total number of viable cells (HVFF + DR‐LSCC) was in a decreasing trend in both groups of DTX‐chitosome (27%) only and MTF/DTX‐chitosomes (15%) but not for other non‐capsulated drug groups (MTF: 85%, DTX: 68%, MTF/DTX: 55%; *p* < 0.05) (Figure 7c). At Day 5, LIVE/DEAD staining for each cell type further confirmed that the MTF/DTX‐chitosome group showed more cell death in both stromal and cancer cells, compared to the non‐capsulated MTF‐DTX group and no treatment controls (Figure 7d). The uptake of fluorescent DTX‐loaded chitosomes by HVFF and DR‐LSCC (Figure 7e), as well as by non‐resistant LSCC, was qualitatively observed via immunostaining, both suggesting similar uptake regardless of the presence or absence of the drug.

## 3 DISCUSSION

Acquired drug resistance is a significant challenge to laryngeal cancer treatments. One strategy might involve the use of chemosensitizers like MTF to enhance the efficacy of chemotherapeutic agents by inhibiting the mTOR pathway.^90^ The lack of a preclinical in vitro model of chemoresistant laryngeal cancer has, however, impeded the progression of testing new treatment strategies for restoring tumor sensitivity. Using a stepwise intermittent protocol, we successfully developed a DTX‐resistant laryngeal cancer cell model as shown by distinct gene expression. Genes were downregulated with regards to mucous expression (MUC15), microtubule (FRY and KNL1), apoptosis (TP53, TNFRSF11A, and BCL2), proliferation (MKI67), metabolism (IRS1 and SGK1), endocytosis (CAV1/caveolin), metastasis (SLC16A1 and COL4A1), and chemotherapy‐induced senescence (BIRC5). However, our main focus of the study was the upregulation of genes associated with ABC transporters (ABCC3), mucosa glycosylation (MUC1, MUCL1, MUC2, MUC4, MUC5AC, and MUC17), endocytosis (PIP5K1, UBR3, and FOLR1), microtubules (TUBB3 and ALK), receptors (PDGFR, NOTCH1), metabolism (PIK3CA, DEPTOR, CASTOR1, GSK3B, and CYP24A1), hypoxia (HIF1A), apoptosis (NTRK1, MDM2), cytokine production (STAT1, IL6, and GM‐CSF/CSF2RA) and autophagy (CFLAR‐AS1, ATG13, and ULK1), and chemotherapy‐induced senescence (FGRF1 and IGF1R).

DTX resistance influences the upregulation of ABC transporters, microtubule dynamics, and taxane metabolism.^70^, ^91^, ^92^ ABC transporters, like P‐glycoprotein encoded by ABCB1, are commonly known as multidrug resistance protein 1.^17^, ^20^, ^21^ These efflux transporters play critical roles in reducing the intracellular accumulation of DTX by pumping the drug out of cancer cells.^17^, ^20^, ^21^ In addition to ABCB1, increased resistance to DTX is correlated to an upregulated expression of the ABCC3 ATP binding cassette gene, which ultimately diminishes the efficacy of cancer treatment.^44^, ^93^ DTX hampers microtubule dynamics by binding to the β‐subunit of microtubules like β‐III tubulin, which causes inhibition of mitotic processes.^70^ Upregulated β‐III tubulin with TUBB3 as an encoding gene is associated with reducing the DTX‐related mitotic arrest on microtubule dynamics, leading to treatment resistance.^17^, ^18^, ^19^ With regards to DTX drug metabolic breakdown, its enzymatic degradation relates to CYP family.^17^ CYP24A1 is a CYP family gene that encodes 24‐hydroxylase enzymes helping regulate vitamin D activity.^58^ Upregulated CYP24A1 is associated with DTX resistance,^55^ and poor overall survival of oral cancer patients.^58^, ^59^

Our overall findings are corroborated with other studies in HNC,^26^, ^45^ and DTX‐related studies in cancer (Table S2). For example, with regards to DTX‐resistance genes in pancreatic cancer, the drug efflux ABBC3,^91^ metabolic PIK3CA^94^ and CYP24A^92^ were upregulated in our DR‐LSCC compared to their non‐resistant counterparts. Our transcriptomic data revealed additional genes of mucin MUCL1, MUC4, MUC15 and MUC17, endocytic UBR3, mitotic FRY, metabolic CASTOR1, IRS1, and SGK1, apoptotic NTRK1, metastatic SLC16A1, and autophagic CFLAR‐AS1, ATG13 and ULK1 that were mutated in our DTX‐resistant laryngeal cancer cells, which have not been identified to be associated with DTX resistance in other types of cancer before.

Besides, genes from molecular pathways of autophagy (ULK/ATG complex), drug efflux (ABC transporters), mitochondrial activity (V‐ATP synthase), hypoxia (HIF1A), and PI3K/mTOR (PIK3CA, CASTOR1, and DEPTOR) were upregulated in resistant laryngeal cancer cells. Autophagy maintains cellular homeostasis by degrading intracellular molecules and damaged organelles, which have an impact on drug degradation once internalized by cancer cells.^95^ Increased autophagy activity protects cells from the therapeutic effects of drugs as shown by our resistant laryngeal cancer cell model. Another common contributor to chemoresistance is the upregulation of the ABC transporters, like ABCC3, whose role is to export drugs out of the tumor cells via transmembrane ATP pumps.^96^ Such a drug efflux process diminishes drug cytotoxic effects due to the remaining scarce drug concentration inside cells. Thus, the highly expressed transmembrane transporter and symporter activities may reflect the increased drug efflux of the resistant laryngeal cancer cell model and its associated cytotoxic resistance to drug‐free forms.

Regarding cellular senescence, DR‐LSCC exhibited significantly downregulated BIRC5 and upregulated FGFR1 gene transcription in comparison to non‐resistant LSCC. Cellular senescence is an irreversible cell cycle arrest, which is provoked after cellular stresses like successive chemotherapy treatments.^61^, ^62^ Specifically with a DTX regimen, depletion of survivin (encoded by BIRC5) was reported in cervical cancer cells responding to the taxane treatment.^60^ Taxane‐induced apoptosis was hindered during the mitotic phase of the spindle assembly checkpoint.^60^ In HNC, survivin/BIRC5 was reported to play a role in cancer recurrence associated with radioresistance^97^ and hypoxia‐induced multidrug resistance.^98^ Meanwhile, FGFR1 is another transmembrane receptor that contributes to regulating cellular senescence.^99^, ^100^ In HNC, FGFR1 was reported to be overexpressed in tissue samples from patients with LSCC compared to those with normal laryngeal mucosa.^101^ Targeting senescence‐related genes with chemosensitizers (e.g., MTF^102^) or senolytic agents (e.g., ABT‐263/Navitoclax^103^) can thus be exploited for anti‐cancer therapy in HNC (Table S2, Figure S10).

For the treatment of DTX‐acquired resistance, the proposed encapsulation of DTX into mucoadhesive chitosan‐coated liposomes^13^ could improve drug bioavailability inside and surrounding cancer cells that potentially enhance drug sensitivity via mucin‐chitosan interaction. One issue with low therapeutic effect of the anti‐cancer drugs, particularly for HNC, is related to the route of drug administration. Systemic intravenous delivery of chemotherapy induces highly toxic effects throughout the body because the chemotherapy concentration in the tumor site is like that found in the entire body, with only approximately 1% of the chemotherapy reaching solid tumors via this route.^104^, ^105^ Although locoregional intra‐arterial chemotherapy is proposed as a means of mitigating systemic toxicities by directing the drug into tumor‐supplying arteries rather than through systemic circulation, this administration still carries the risk of causing toxic extravasation damage in the surrounding tumor region.^13^, ^106^ In our previous work, we encapsulated DTX into anionic liposomes coated with cationic chitosan, that is, mucoadhesive chitosomes, for quasi‐targeted local controlled and sustained release of drugs.^13^ We then administered DTX‐loaded chitosomes to non‐resistant mucosal LSCC targeted via mucin‐chitosan interactions. After a week, DTX‐loaded chitosomes exhibited about 20% more laryngeal cancer cell death compared to DTX alone.^13^

The mucin‐related genes, MUC1 and MUC4, are overexpressed in treatment‐resistant laryngeal carcinoma.^107^ DR‐LSCC showed over 1‐log2 fold increase in multiple mucin‐related genes (e.g., MUC1, MUCL1, MUC2, MUC4, MUC5A, and MUC17) compared to both non‐resistant controls. However, DR‐LSCC exhibited a non‐significant 0.2‐log2 fold downregulation of MUC15 compared to non‐resistant LSCC. As noted, downregulated MUC15 expression is associated with poor clinical prognosis in hepatocellular carcinoma.^108^ Anionic carboxyl groups in mucins, that is, MUC1 encoding Mucin 1 transmembrane protein, interact electrostatically with the cationic amine groups in chitosan, which forms protein–polysaccharide complexes.^109^ In our previous study,^13^ cationic chitosome mucoadhesiveness was confirmed by turbidimetry and absorbance readouts using an anionic Mucin 1‐riched dispersion. In turn, DTX‐loaded chitosomes exhibited increased cytotoxic effects in LSCC compared to non‐encapsulated DTX exposure. Our chitosome system could be compatible with the local delivery of DTX to mucin‐overexpressing tumors such as by intra‐arterial delivery, that is, a drug to be delivered into tumor's supplying artery.^44^ Intra‐arterial delivery can help limit deleterious systemic side effects and likely target metastatic tumor cells, especially for locally advanced laryngeal cancer, that is, stages III and IV. We anticipate that by targeting mucin‐overexpressing laryngeal cancer cells, the chitosan‐coated liposomes will electrostatically bind to transmembrane mucins like MUC1, MUC4, MUC17 and thus enhance the drug intake and retention process.^108^, ^109^

Encapsulated drugs are often internalized by cells via endocytosis mechanisms rather than transmembrane transporter activity.^110^ In endocytosis, the internalized substance interacts with cell membrane via plasma membrane vesicles. For nanoparticle carriers like liposomes, the related endocytosis processes are still in debate that may relate to dependent or independent mechanisms of the transmembrane protein clathrin.^111^ In our study, DR‐LSCC exhibited upregulation of the PIP5K1A (Table S2), an upstream PI3K/mTOR pathway actuator^112^ present in clathrin endocytic mechanisms. Interestingly, both clathrin‐dependent gene (UBR3) and clathrin‐independent gene (FOLR1) were also upregulated in DR‐LSCC, suggesting that chitosomes may be uptaken primarily via clathrin‐dependent endocytic processes since the caveolin gene (CAV1) related to the clathrin‐independent mechanism was downregulated by DR‐LSCC (Table S2).

In our chemosensitizing study, DR‐LSCC primed with MTF showed higher cell death than non‐resistant LSCC in 2D cultures. To demonstrate the physiological relevance of our DR‐LSCC model, a resistant tumor‐on‐a‐chip was developed to emulate the hypoxic tumor core by co‐culturing laryngeal fibroblast embedded in a collagen gel with laryngeal cancer cells. Compared to DR‐LSCC, stromal cells showed ~10% more cell death after treatment of encapsulated drugs. This result may be attributed to the fact that the stromal chamber had greater exposure to the drug agents because of its immediate proximity to the drug‐media channel. Another explanation could be owing to the sustained and controlled released of DTX from the mucoadhesive chitosomes^13^ entrapped within the microfluidic device. This chip design may have trapped the chitosomes within the microfluidic device. Non‐encapsulated DTX, MTF, and MTF/DTX treatments showed a ~15% recovery in cell viability considering the co‐culture cell number on Day 5, which is attributable to drugs being washed away with less than 40% retention rate. Low drug retention gave time for the cells to recover from the treatment's cytotoxic effects. Interestingly, such recovery in cell viability was not observed in the treatment groups of the DTX‐loaded chitosomes with or without MTF.

For this very first laryngeal‐tumor‐on‐a‐chip, the hypoxic‐driven setups corroborated the interplay between increased oxidative stress and reduced oxygen levels.^113^ DR‐LSCC was housed in a presumably less oxygen tension than HVFF in the chip. Interestingly, DR‐LSCC showed strong intracellular hypoxia but low oxidative stress signals, whereas HVFF showed strong signals in these two intracellular markers. This observation may be likely caused by the antioxidant behavior of DR‐LSCC, marked by their dysfunctional mitochondrial activity leading to an increased GLRX expression that consequently regulates intracellular hypoxia (HIF‐1, nitroreductase).

As noted in lung cancer cells, the antioxidant gene of GLRX, a regulator of the HIF‐1,^114^ is associated with treatment resistance, in which their overexpression may result in treatment failure.^115^ Also, chemoresistance‐associated mitochondrial oxidative phosphorylation^116^, ^117^, ^118^ may implicate the oxidative stress and intracellular respiratory processes in DR‐LSCC. From our transcriptomic findings, DR‐LSCC showed upregulated MT‐ND1 and downregulated mitochondrial‐related genes including ATP5PO, ATP5F1B, CYC1, SDHA, NDUFA4, and COX6B1. In this case, increased GLRX expression and impaired mitochondrial activity may collectively regulate the oxidative and hypoxic behavior (i.e., increased HIF1A) exhibited by the resistant laryngeal cancer cells (Figure S10).

Further work is warranted in employing this cell culture model for chemoresistant drug testing. Here, the gene stability of DR‐LSCC was confirmed with the test of one freeze–thaw cycle. However, additional freeze–thaw cycle tests could be performed to evaluate if resistance would be preserved in this cell line. In addition to UM‐SCC‐17A used in this study, other laryngeal or HNC cell lines (e.g., Hep‐2^119^ and MDA1586^120^) can be subjected to the same drug induction regimen for reproducibility evaluation.

In the current MTF‐sensitization experiment, hypoxia analysis was only performed qualitatively via fluorescence staining on microfluidic chip cultures at best. Semi‐quantitative methods like qPCR and phosphorescence lifetime imaging could be used to interrogate hypoxia for short‐term/real‐time detection of O_2_ gradients at a higher resolution.^121^ Meanwhile, retrieving RNA samples from on‐chip cultures for robust transcription analysis remains an unresolved challenge in the field. Current advances in on‐chip RNA isolation^122^ and spatial transcriptome sequencing^123^ could be explored for more in‐depth spatiotemporal analysis of hypoxia‐cell interactions in tumor‐on‐a‐chip studies.

Lastly, DR‐LSCC was co‐cultured with stromal HVFF in the tumor‐on‐a‐chip experiment. Further work could include immune cells, such as tumor‐associated macrophages, in the culturing system for enhanced in vivo representation. Tumor‐associated macrophages are known for being tumor scavenger/immunosuppressive cells.^124^ DTX was reported to promote monocyte polarization into the tumor‐associated phenotype as part of acquiring DTX resistance, resulting in poor clinical prognosis and cancer recurrence.^124^, ^125^ Besides, the ECM environment of a laryngeal tumor can be better matched to the dense, stiff stroma of in vivo laryngeal tumors, which is crucial for the evaluation of liposome/nanocarrier sequestration.^104^ For instance, decellularized ECM crosslinked hydrogels could be tuned to resemble the mechano‐phenotype of laryngeal tumors and be used as cell substrates in the chip system.^104^, ^126^, ^127^, ^128^, ^129^, ^130^ To fully decipher the complex interactions of hypoxia and drug response (uptake, sensitivity and resistance), tri‐cultures (cancer‐stroma‐macrophage) and tumor‐like cell substrates would be two key elements to be incorporated in future laryngeal‐tumor‐on‐a‐chip culturing system.

## 4 CONCLUSION

In sum, chemoresistance is an incalcitrant clinical challenge in laryngeal cancer treatment. A physiology‐representative in vitro model is warranted to decipher critical molecular mechanisms associated with chemoresistance. We developed a chemoresistant laryngeal cancer cell model whose genotypic and phenotypic profiles were collaborated to those known in the literature as HNC and other cancer studies. This resistant cell model can be applied to help elucidate tumor‐stromal interaction in chemoresistance and evaluate new drug strategies in the reduction of chemoresistance for laryngeal cancer.

## 5 EXPERIMENTAL METHODS

### 5.1 Cell Culture

Three cell lines were used for this study: (1) A non‐chemoresistant human laryngeal cancer cell line (LSCC) isolated from primary laryngeal carcinoma located at the supraglottis in T2 or T3 stage assumable^131^, ^132^ of a 48‐year‐old female patient who did not benefit from radiotherapy; (2) a human immortalized vocal fold fibroblast cell line (HVFF)^133^ representing stromal cells in the laryngeal tumor; and (3) a human laryngeal epithelial cell line (LSEC) representing healthy laryngeal epithelia. LSCC and HVFF cell lines were grown in LSCC complete media consisting of high glucose DMEM, 10% FBS, 1% non‐essential amino‐acids and 1% penicillin/streptomycin in a humidified atmosphere of 5% CO_2_ at 37°C. LSEC were grown in ATCC dermal cell basal media. After reaching 70%–80% confluency in T‐75, cells were cultured in fresh Free‐FBS media for 1 day to synchronize cell cycle, and were then harvested using TrypLE for 5–15 min. After adding fresh media, cells were counted by a hemocytometer before being centrifuged at 900 rpm for 5 min. Media was discarded and cells were resuspended in fresh media with a working concentration of 1 × 10^6^ cells/mL. Both HVFF and LSEC between passages 3–5 were used for this study.

### 5.2 Experimentally inducing cell resistance to DTX drugs

LSCC were monthly exposed to escalating doses of DTX. LSCC were exposed to increasing doses (50 nM, 100 nm, 200 nM, and 400 nM) for up to 4 months to induce the DTX‐resistant phenotype on cancer cells. LSCC were exposed to DTX‐enriched LSCC media for 3 days followed by a 3‐day exposure of regular LSCC media. A series of experiments was performed on DR LSCC after the escalating 4‐month exposure of DTX (Table 1).

**TABLE 1:** Key resources.

Reagent	Source	Identifier
Materials for transcriptomic and functional analysis		
RNeasy Micro Kit (50)	QIAGEN, Toronto, Canada	74,004
TruSeq Stranded Total RNA kits	Illumina, San Diego, USA	20,020,597
Qubit RNA HS Assay	Thermo Fisher Scientific, Waltham, USA	Q32855
High‐sensitivity RNA screen tape	Agilent, Santa Clara, USA	5067–5579
qPCR Library quantification kit	Roche Diagnostics, Laval, Canada	7,960,140,001
mTOR ELISA kit	Abcam, Cambridge, UK	ab279869
LDH assay kit	Abcam, Cambridge, UK	ab65393
ADP/ATP assay kit	Abcam, Cambridge, UK	ab83359
Autophagy kit	Abcam, Cambridge, UK	ab133075
MMP‐3 Activity Assay Kit	Abcam, Cambridge, UK	ab118972
DAPI	Abcam, Cambridge, UK	ab228549
ALEXA647/EGFR	Abcam, Cambridge, UK	ab192982
ALEXA488/TUBIII	Abcam, Cambridge, UK	ab195879
ALEXA594/PI3KCA	Abcam, Cambridge, UK	ab282113
ALEXA647/p53	Abcam, Cambridge, UK	ab224942
ALEXA555/αSMA	Abcam, Cambridge, UK	ab202509
ALEXA647/Vim	Abcam, Cambridge, UK	ab194719
ALEXA488/CK5	Abcam, Cambridge, UK	ab193894
eFluor615/ki‐67	Thermo Fisher Scientific, Waltham, USA	42–5698‐82
ALEXA488/OCT3/4	Thermo Fisher Scientific, Waltham, USA	53–5841–82
LIVE/DEAD staining	Thermo Fisher Scientific, Waltham, USA	L3224
MTT assay	Thermo Fisher Scientific, Waltham, USA	V13154
ROS‐ID® Hypoxia/Oxidative stress detection kit	Enzo life sciences, NY, USA	ENZ‐51042‐0125
DAPI	Abcam, Cambridge, UK	ab228549
Materials for cell culture experiments		
LSEC	ATCC, Manassas, USA	CRL‐3342
Dermal Cell Basal Medium	ATCC, Manassas, USA	PCS‐200‐030
LSCC	MilliPore‐Sigma, Burlington, USA	UM‐SCC‐17A
HVFF	University of Wisconsin‐Madison	Thibeault Lab
Collagen I	Thermo Fisher Scientific, Waltham, USA	A1048301
Mucin 1 powder	MilliPore‐Sigma, Burlington, USA	M3895
BEGradient Barrier Free	BEOnChip, Zaragoza, Spain	1,000,320
Pooled Human Plasma	Innovative Research Inc., Novi, USA	IPLAWBNAE50ML
8‐chamber slides	Lab‐Tek®II	154,534
Docetaxel	MilliPore‐Sigma, Burlington, USA	PHR1883
Metformin	MilliPore‐Sigma, Burlington, USA	PHR1084
Materials for chitosome experiments		
Cholesterol	MilliPore‐Sigma, Burlington, USA	C8667
DSPC	MilliPore‐Sigma, Burlington, USA	850365P
DSPE‐PEG2000	MilliPore‐Sigma, Burlington, USA	880120P
Liss Rhod PE	MilliPore‐Sigma, Burlington, USA	810150P
Pacific Hemostasis™ APTT	Thermo Fisher Scientific, Waltham, USA	100,309
Equipment/Software		
Zeiss Axiover3	Zeiss, Germany	Florescence microscope
Spectramax i3	Molecular Devices, San Jose, USA	Plate reader
LightCycler® 480	Roche Diagnostics, Laval, Canada	4,729,749,001
DNA High Sensitivity LabChip	PerkinElmer, Waltham, USA	CLS760672
Tecnai G2 F20 200 kV	Fei, Hillsboro, USA	TEM
Imaris version 9.5.1 Software	Bitplane, South Windsor, CT	Image processing software
BioRender	BioRender, Toronto, Canada	Scientific illustration software
GraphPad Prism version 9.5.1	GraphPad Software, San Diego, CA, USA	Scientific data analysis software

### 5.3 RNA‐sequencing of transcriptomic profiles

RNA‐sequencing analysis was performed at the McGill Genome Centre. Transcriptomic profiling was performed on three cell groups cultured in conventional 2D flat monolayers: (1) the DR laryngeal cancer cells (DR‐LSCC), (2) non‐treated laryngeal cancer cells (LSCC), and (3) non‐treated laryngeal epithelial cells (LSEC). The RNeasy® mini Kit was used to isolate and purify the RNA from cells according to the manufacturer's guidelines. Such an analysis was carried out on LoBind tubes containing isolated RNA. A total of 5 replicates per group × 3 cell groups = 15 in total, and 150 M reads per sample were conducted. For the initial RNA quality check, concentrations were measured using the Qubit RNA HS Assay. Then, the RNA integrity profile was verified using a High sensitivity RNA screen tape. RNA libraries were constructed using the TruSeq Stranded Total RNA from Illumina following the manufacturer's protocol. Library QC was measured by qPCR with the library quantification kit and LightCycler® 480 from Roche Diagnostics. Finally, the library profile was measured with a DNA High Sensitivity LabChip. Only samples that met the RNA quantity and quality concentration of about 150 ng/L were subjected to subsequent sequencing (Table S1).

### 5.4 RNA‐sequencing data analysis

RNA sequencing was processed using the TruSeq Stranded Total RNA With Illumina Ribo‐Zero Plus rRNA Depletion workflow to obtain the LibQC results. Adaptor sequences and low‐quality score bases (Phred score < 30) were first trimmed using Trimmomatic.^134^ The resulting reads were aligned to the GRCh38 human reference genome assembly, using STAR.^135^ Read counts were obtained using HTSeq^136^ with parameters ‐m intersection‐nonempty‐stranded = reverse. For all downstream analyses, we excluded lowly‐expressed genes with an average read count lower than 10 across all samples. Raw counts were normalized using edgeR's TMM algorithm^137^ and were then transformed to log2‐counts per million (logCPM) using the voom function implemented in the limma R package.^138^ To assess differences in gene expression levels, we fitted a linear model using limma's lmfit function. Nominal *p*‐values were corrected for multiple testing using the Benjamini‐Hochberg method. GSEA based on a pre‐ranked gene list by t‐statistic was performed using the R package fgsea (http://bioconductor.org/packages/fgsea/). In GSEA, the scoring process was repeated 1000 times and the *p*‐value was determined by comparing how frequently the Enrichment score from the actual ranking surpassed that obtained from random permutations. Padj values were computed by evaluating the distribution of Normalized Enrichment Scores across numerous gene sets. KEGG pathway diagrams were generated using the visualization tools Pathview^139^ and KEGGscape.^140^ The RNA sequencing data generated in this study have been deposited to the National Center for Biotechnology Information Gene Expression Omnibus (GEO) with accession codes GSE248302.

### 5.5 Cell cytotoxicity

Cell viability assay was performed on LSEC, LSCC, and DR‐LSCC with approximately 1 × 10^4^ cells seeded separately on 8‐chamber slides. After reaching 100% confluency, 1 μM DTX was added to the culture media in each of the slide's chambers. On Day 3, cells were washed with 1× PBS before being stained, using a LIVE/DEAD viability/cytotoxicity assay kit with green‐fluorescent calcein‐AM and red‐fluorescent ethidium homodimer‐1 dyes following the manufacturer's instructions. The slides were incubated for 30 min in darkness at room temperature before being washed twice with 1× PBS. Zeiss Axiovert3 inverted fluorescence microscope with 10× objective was used to acquire cell images with FITC (LIVE, green) and Cy3 (DEAD, red/orange) filters.

MTT analyses were also carried out to obtain quantitative data on cell viability. For this assay, about 5 × 10^3^ cells were seeded in individual 96 well plates following manufactures' guidelines. A Spectramax i3 plate reader was used to determine the absorbance of MTT at λ = 570 nm. Percentage of cell viability was calculated using Equation (1).

(1) $$\text{Cell viability}\left(\%\right)=\frac{\left(\text{Non}-\text{treated control}\right)-\left(\text{treated celss}\right)}{\text{Non}-\text{treated control}}\times 100$$

The therapeutic effect of MTF on cells was determined using LIVE/DEAD staining and MTT readouts to obtain qualitative and quantitative cell cytotoxicity data. First, the effect of MTF (500 μM, 1, 5, and 10 mM) was assessed on HVFF, LSEC, LSCC and DR‐LSCC after 3 days of exposure. Second, cytotoxic effect of the combination therapy MTF/DTX‐loaded chitosomes was evaluated for up to 7 days on the same groups and freeze‐thawed DR‐LSCC. Lastly, the morphology of the DTX‐loaded chitosomes was examined by transmission electron microscopy using a FEI Tecnai G2 F20 transmission electron microscope (TEM) at a voltage of 120 kV. The coagulation effect of DTX‐loaded chitosomes was evaluated using the activated partial thromboplastin time (APTT) method^141^ up to 15 min exposure.

### 5.6 Autophagy analysis

Autophagy analysis was performed on LSEC, LSCC, and DR‐LSCC following similar experimental conditions as that of the cell cytotoxicity analysis described above. By following manufacturer's protocol, we discarded the media and added 100 μL of the Cell‐Based Propidium Iodide solution in each well and cells were incubated for 2 min at room temperature. A washing step was proceeded with 100 μL of Cell‐Based Assay Buffer. A 100 μL of the Cell‐Based MDC solution was then added to each well and cells were incubated for 10 min at 37°C. After another washing step, cells were imaged with fluorescent microscopy at 63× magnification with Zeiss Axiovert3. 10 μM tamoxifen was used as a positive control. Dead cells were stained by propidium iodide and detected with a Texas Red filter. Autophagic vacuoles were stained by Monodansylcadaverine (MDC) and detected with a DAPI filter. Absorbance readouts were conducted using the Spectramax i3 plate reader with UV‐DAPI and Texas red wavelengths as specified by manufacturer guidelines.

### 5.7 Immunocytochemistry analysis

DR‐LSCC, LSCC, and LSEC were fixed with 4% paraformaldehyde in PBS for 10 min at room temperature. Then cells were blocked with 0.1% Triton X‐100 in PBS (blocking solution) for 30 min at room temperature. Followed by blocking with 10% normal goat serum with 0.5% Triton X‐100 in PBS (blocking solution) for 30 min at room temperature. Fluorochrome‐conjugated primary antibodies with a 1/150 ratio: ALEXA488/ TUBIII, eFluor615/ki‐67, ALEXA647/EGFR, ALEXA488/OCT3‐4, ALEXA594/PI3KCA, ALEXA647/p53, ALEXA488/ECAD, ALEXA555/αSMA, and ALEXA647/Vim were diluted in blocking solution and incubated at 37°C for 1 h. DAPI was applied for 10 min at room temperature. The cells were then imaged with 10x magnification using the Zeiss Axiover3 using filters corresponding to the conjugated antibody and retrieved using Imaris version 9.5.1 Software.

### 5.8 mTOR ELISA

Expression of pan‐mTOR and phospho‐mTOR was analyzed on DR‐LSCC, LSCC, LSEC, and ELISA was used to quantify the intracellular mTOR expression. In brief, cells were washed with PBS, followed by adding the lysis buffer. Cells were solubilized at about 4 × 10^7^ cells/mL in prepared Cell Lysate Buffer by gently pipetting up and down to resuspend the pellet. Lysates were incubated with shaking at 4°C for 30 min. Microcentrifuge at 13,000 rpm for 10 min at 4°C and transfer the supernatants into a clean test tube. Lysates were 80‐fold diluted with Assay Diluent. We continued following manufacturer's guidelines and read at 450 nm using the Spectramax i3 plate reader.

### 5.9 MMP‐3 activity assay

MMP‐3 Activity Assay was performed to measure MMP‐3 activity in cell culture media (secreted protein, 40‐fold diluted) and cell lysates (intracellular protein, 100‐fold diluted) from DR‐LSCC, LSCC, and LSEC at 4 × 10^7^ cells/mL. To directly measure MMP‐3 activity,^142^ cells (1 × 10^6^) were homogenized in 200 μL ice‐cold MMP‐3 Assay Buffer then centrifuged to remove insoluble material at 13,000 × g for 10 min. Sample volumes of 50 μL/well were mixed with Assay Buffer in a 96‐well plate. After reacting with the MMP‐3 substrate (prepared according to the protocol), the plate was read at Ex/Em = 325/393 nm twice in 2 h using the Spectramax i3 plate reader. Read R1 at T1. Read R2 again at T2 after incubating the reaction at room temperature for 60 min, protected from light. The RFU of fluorescence generated by hydrolyzation of the substrate is (2):

(2) ΔRFU=R2–R1

### 5.10 ATP/ADP assay

To evaluate metabolic activity, intracellular ATP/ADP ratios were measured via ATP and ADP assays. DR‐LSCC, LSCC, and LSEC at 4 × 10^7^ cells/mL were washed twice in cold PBS before harvesting and diluted 80‐folds. Cells were resuspended and homogenized in 100 μL of Assay Buffer IV/ADP Assay Buffer. Then, cells were incubated on ice for 10 min. Samples were centrifuged for 5 min at 4°C at top speed to remove any insoluble material. Supernatants were collected and kept on ice. 50 μL of Reaction Mix was added to each standard, sample, and background control wells. The plate was incubated at room temperature for 30 min protected from light, followed by measuring the output using the Spectramax i3 plate reader at OD 570 nm.

### 5.11 Clonogenic assay

Colony formation was analyzed after exposure to DTX alone, DTX‐loaded liposomes, and DTX‐loaded chitosomes, LSCCs were seeded in 24 well‐plates at a density of 15 × 10^3^ and incubated until full confluency up to 7 days. After treatments, the culture medium was removed, and the cells were washed twice with PBS. Then, cells were stained with 0.1% crystal violet (water 30%, ethanol 70%) in sterile water (0.5 mL/well) for 30 min at room temperature. After thorough washing, the colonies were analyzed via images taken with a cellphone camera.

### 5.12 Microfluidic analyses

The microfluidic device Gradient Barrier‐Free from BEOnChip (Zaragoza, Spain) was used for this analysis because of the desired representation of hypoxic tumor models. The device comprised two lateral channels, referred to as drug‐media and cancer channels in this study, interconnected via a central chamber referred to as stromal chamber. For cell seeding, microfluidic devices were warmed at 37°C for 24 h, to prevent massive bubble formation. HVFF were embedded in a 2 mg/mL collagen I gel following the gel preparation protocol of the manufacturer. After encapsulating HVFF in the collagen I gel, 7 μL of HVFF‐containing gel was injected into the central chamber (stromal chamber). The central chamber inlet and outlet were sealed with adhesive tape provided by the BEOnchip. After 4 h, cancer channel was coated with 0.5 mg/mL oral mucin 1 solution to emulate a mucosal tumor as in vivo.^86^ After 1 h, one wash with culture media was performed before injecting 15 μL cancer cells into the mucin‐coated lateral channels (cancer channel). Cancer channel inlet and outlet were also sealed with plugs to create a diffusion gradient. After 1 h, culture media was infused in the remaining lateral channel (drug‐media channel) at a 0.65 μL/min rate simulating interstitial flow using an Ismatec peristaltic pump (Figure S11, Video S1). The whole system was placed inside the Biosafety Cabinet to maintain sterile conditions.

### 5.13 Functional verification of tumor‐on‐a‐chip

#### 5.13.1 Phenotypic markers and migration assay

DR‐LSCC and HVFF were expected to overexpress TUBIII^13^ and vimentin,^143^ respectively. Phenotypic markers were evaluated at Day 0 which occurred 24 h after seeding cells into the microfluidic device with immunostating of ALEXA488/TUBIII and ALEXA647/Vim. Migration of HVFFs into the cancer channel was assessed after 1, 3, and 5 days. Controls comprised 4 h exposures of (1) 10 mM MTF to hinder hypoxia/proliferation and (2) 200 μM deferoxamine to induce hypoxia/proliferation, respectively. Cells were imaged by 40× magnification using the Zeiss Axiovert3 using ALEXA488 and ALEXA647 filters and retrieved using Imaris version 9.5.1 Software.

#### 5.13.2 Hypoxia/oxidative stress assay

Nitroreductase activity in hypoxic cells was measured using a hypoxia detection kit. Briefly, DR‐LSCC and HVFF microfluidic cultures were evaluated during Day 0 at 6, 12, and 24 h. Deferoxamine (200 μM, hypoxia inducer) and Pyocyanin (200 μM, oxidative stress inducer) were used as a positive control after 4 h exposure. Cells were washed twice with PBS, and incubated with the hypoxia/oxidative stress detection mix for 30 min. Next, the detection mix was discarded, and cells were washed twice with PBS. Cells were imaged by 63× magnification using the Zeiss Axiovert3 with ALEXA488 and ALEXA594 filters and retrieved using Imaris version 9.5.1 Software.

### 5.14 Microfluidic evaluation of combined MTF/DTX strategy

The set‐up of the chemosensitivity study was to mimic a 12 min intra‐arterial injection of chemotherapeutics^144^, ^145^ in vitro. A treatment of 1 mM MTF was administered for 1 day continuously flowing in the drug‐media microchannel. After the first 6 h of MTF chemosensitizing infusion, 1 μM DTX‐loaded chitosomes (about 1 × 10^8^ nanocarriers) or 1 μM DTX were infused once for 12 min in the drug‐media channel at 5 μL/min to match the interstitial tumor injection rates.^146^ Then, MTF was infused for the rest of the day, that is, 18 hrs. Drug‐free media was flown afterwards until the end point of the study.

Controls of 1 mM MTF, 1 μM DTX alone, and 1 μM DTX‐loaded chitosomes on DR‐LSCC and HVFF were evaluated at Day 0 (100% confluent control) and time points for 1, 3, and 5 days in the co‐culture setup. DAPI staining was used to image the cells after treatments at 10× magnification using the Zeiss Axiovert3 and Imaris version 9.5.1 Software. For cell counting, cell nuclei (10 μm) in two regions of interest (stromal chamber and cancer channel) were counted using the spot detection algorithm and DAPI mean fluorescence intensity. LIVE/DEAD staining was adapted from Section 5.5 to evaluate the combination therapies (MTF/DTX, and MTF/DTX‐loaded chitosomes) in the co‐culture setup at Days 0 and 5. Zeiss Axiovert3 inverted fluorescence microscope with 40× objective was used to acquire cell images with FITC (LIVE, green) and Cy3 (DEAD, red/orange) filters.

In addition, an LDH assay was performed to analyze cytotoxicity and verify the qualitative DAPI images. Supernatant was collected from drug‐media outlets at 6, 12, 24, 60, and 360 min after starting infusing nanoparticles (a 12‐min injection). LDH percentages of cell viability were calculated using equation (1). Moreover, fluorescent chitosomes uptake^13^ after 6 h inspection with the inverted microscope Zeiss Axiovert 3. DR‐LSCC was anticipated to express EGFR considering that LSCC is known to stain positively with EGFR (ALEXA647/EGFR).^147^, ^148^ Considering the 24‐hr co‐culturing, HVFF was also expected to exhibit a transition to cancer‐associated fibroblast phenotype with positive EGFR stain.^88^, ^89^ Cytokeratin 5 (ALEXA488/CK5) is a squamous differentiation marker and is exclusively expressed on DR‐LSCC.^149^ Cytokeratin 5 would thus be used to differentiate DR‐LSCC (CK5^+^) from HVFF (CK5^−^).

### 5.15 Statistical analysis

The data are reported as mean ± SD. The statistical significance of the differences was analyzed by one‐way or two‐way ANOVA (all assumptions were met) and Bonferroni as a post hoc test to assess differences between pair groups using GraphPad Prism version 9.5.1.

## AUTHOR CONTRIBUTIONS

C.R.M.‐G.: conceptualization, data plotting, visualization, writing‐original draft, and writing‐reviewing and editing. M.M: reviewing and editing. A.P: data plotting, visualization, reviewing and editing. N.S: reviewing and editing. M.T.: conceptualization, visualization, writing‐original draft, reviewing and editing, supervision, funding acquisition, and project administration. N.Y.K.L.‐J.: conceptualization, visualization, writing‐original draft, writing‐reviewing and editing, supervision, funding acquisition, and project administration.

## FUNDING INFORMATION

This study was supported by the National Sciences and Engineering Research Council of Canada (RGPIN‐2018–03843, RGPIN‐2024‐04235 and ALLRP 548623‐19), Canada Research Chair research stipend (M.T. and N.Y.K.L.‐J.) and the National Institutes of Health (R01 DC‐018577‐01A1). The presented content is solely the responsibility of the authors and does not necessarily represent the official views of the above funding agencies.

## CONFLICT OF INTEREST STATEMENT

The authors declare no conflict of interest.

## Supporting information


Data S1.

Video S1.

## Data Availability

The RNA‐seq data generated in this study are available at the National Center for Biotechnology Information Gene Expression Omnibus (GEO) with accession codes GSE248302. All other included data in this study are available from the corresponding authors upon reasonable request.
