# Supplementary material for: Establishment of a chemoresistant laryngeal cancer cell model to study chemoresistance and chemosensitization responses via transcriptomic analysis and a tumor‐on‐a‐chip platform
Source: Bioeng Transl Med. 2025 Jan 22;10(3):e10741. doi: 10.1002/btm2.10741 (PMC12079406; doi:10.1002/btm2.10741)
Supplement: Supplementary file 1 — Data S1. [file BTM2-10-e10741-s002.docx]

#### **Establishment of a Chemoresistant Laryngeal Cancer Cell Model to Study Chemoresistance and Chemosensitization Responses Via Transcriptomic Analysis and a Tumor-On-A-Chip Platform**

Christian R. Moya-Garcia, Meghana Munipalle, Alain Pacis, Nader Sadeghi, Maryam Tabrizian*, Nicole Y. K. Li-Jessen^*^

Christian R. Moya-Garcia

Department of Biomedical Engineering, Faculty of Medicine and Health Sciences, McGill University, 3775 Rue University, Montreal, QC H3A 2B4, Canada

Meghana Munipalle

Department of Biomedical Engineering, Faculty of Medicine and Health Sciences, McGill University, 3775 Rue University, Montreal, QC H3A 2B4, Canada

Alain Pacis

Canadian Centre for Computational Genomics (C3G), Montreal, McGill University, 740 Dr. Penfield Avenue, Room 6103, Montreal, QC H3A 0G1, Canada

Nader Sadeghi

Department of Otolaryngology – Head and Neck Surgery, McGill University, MUHC (Royal Victoria Hospital), 1001 Decarie Blvd., Montreal, Quebec H4A 3J1, Canada

Maryam Tabrizian*

Department of Biomedical Engineering, Faculty of Medicine and Health Sciences, McGill University, 3775 Rue University, Montreal, QC H3A 2B4, Canada

Faculty of Dentistry and Oral Health Sciences, McGill University, 2001 Ave McGill College, Montreal, QC H3A 1G1, Canada

Nicole Y. K. Li-Jessen*

School of Communication Sciences and Disorders, McGill University, 2001 Av. McGill College #8, Montréal, QC H3A 1G1, Canada

Department of Biomedical Engineering, Faculty of Medicine and Health Sciences, McGill University, 3775 Rue University, Montreal, QC H3A 2B4, Canada

Department of Otolaryngology – Head and Neck Surgery, McGill University, MUHC (Royal Victoria Hospital), 1001 Decarie Blvd., Montreal, Quebec H4A 3J1, Canada

Research Institute of the McGill University Health Centre, 1001 Decarie Blvd., Montreal, QC H4A 3J1, Canada

* Equal contribution as senior authors

***** E-mail: [maryam.tabrizian@mcgill.ca](mailto:maryam.tabrizian@mcgill.ca); [nicole.li@mcgill.ca](mailto:nicole.li@mcgill.ca)

Supporting Information

Table S1. RNA quality and quantity of DR-LCC, LSCC, and LSEC.

| Sample Name | Conc. (ng/ul) | Volume (ul) | Total (ng) | Plate coor. | Conc.(ng/ul) dil. 1/5 | Conc.  (ng/ul) | Volume (ul) | Total RNA (ng) | RIN |
| --- | --- | --- | --- | --- | --- | --- | --- | --- | --- |
| DR-LSCC1 | 176.2 | 15 | 2643 | A01 | 12.3 | 61.5 | 13 | 799.5 | 7.9 |
| DR-LSCC2 | 268.8 | 15 | 4032 | B01 | 38 | 190 | 13 | 2470 | 6.3 |
| DR-LSCC3 | 172.3 | 15 | 2585 | C01 | 10.6 | 53 | 13 | 689 | 8.6 |
| DR-LSCC4 | 175.8 | 15 | 2637 | D01 | 37.2 | 186 | 13 | 2418 | 8.2 |
| DR-LSCC5 | 167.5 | 15 | 2513 | E01 | 41.8 | 209 | 13 | 2717 | 8.5 |
| LSCC1 | 475.6 | 15 | 7134 | F01 | 61.6 | 308 | 13 | 4004 | 8.5 |
| LSCC2 | 461 | 15 | 6911 | G01 | 78.6 | 393 | 13 | 5109 | 8.4 |
| LSCC3 | 471 | 15 | 7064 | H01 | 82.6 | 413 | 13 | 5369 | 8.9 |
| LSCC4 | 466 | 15 | 6984 | A02 | 75.4 | 377 | 13 | 4901 | 8.6 |
| LSCC5 | 471 | 15 | 7068 | B02 | 83.6 | 418 | 13 | 5434 | 8.6 |
| LSEC1 | 204 | 15 | 3065 | C02 | 55.2 | 276 | 13 | 3588 | 8.3 |
| LSEC2 | 191 | 15 | 2858 | D02 | 51.8 | 259 | 13 | 3367 | 8.3 |
| LSEC3 | 201 | 15 | 3012 | E02 | 44.8 | 224 | 13 | 2912 | 8.2 |
| LSEC4 | 185 | 15 | 2778 | F02 | 46.8 | 234 | 13 | 3042 | 8 |
| LSEC5 | 166 | 15 | 2487 | G02 | 40.8 | 204 | 13 | 2652 | 9 |

Table S2. Transcriptomic-related altered biological functions associated with docetaxel resistance. Adjusted p-values (*P_adj_*) were based on gene set enrichment analysis (Section 5.4. RNA-sequencing Data Analysis).

| **OUR DATA (LSCC vs. DR-LSCC)** | | | **LITERATURE** | | |
| --- | --- | --- | --- | --- | --- |
| **Altered**  **function** | **Gene** | **Mutation**  **Padj** | **Cancer** | **Chemoresistance**  **Promotion** | **Ref.** |
| Drug efflux | ABBC3 | Upregulated  4.53E-06 | Pancreatic cancer cell lines PC3, DU145, and murine pancreatic cancer cells | PI3K/Akt pathway | 1 |
| Mucous barrier/ glycosylation | MUC1 | Upregulated  0.0006312 | Pancreatic cancer cell lines LNCaP, PC3, and DU145, and xenografts mice models | AR, and cancer stemness pathways | 2 |
|  | MUCL1 | Upregulated  3.54E-08 | Not available | Not available | Not available |
|  | MUC2 | Upregulated  0.003701156 | Patient-derived breast cancer xenograft mice model | Metastasis pathway | 3 |
|  | MUC4 | Upregulated  5.02E-05 | Not available | Not available | Not available |
|  | MUC5AC | Upregulated  1.55E-06 | Gastric cancer clinical samples, in-house cell line, and mice models | Cancer stemness pathways | 4 |
|  | MUC17 | Upregulated  0.000135 | Not available | Not available | Not available |
|  | MUC15 | Downregulated  0.0814225 | Not available | Not available | Not available |
| Endocytosis | PIP5K1A | Upregulated  4.55E-07 | Breast cancer cell line MDA-MB-231, and mice xenograft tumors | PI3K/Akt pathway | 5 |
|  | UBR3 | Upregulated  0.000112078 | Not available | Not available | Not available |
|  | FOLR1 | Upregulated  0.004237585 | Prostate cancer cell lines PC3; C4-2 B and LNCaP | ABC transporters | 6 |
|  | CAV1 | Downregulated  2.52E-10 | Breast cancer cell lines  BT474, Hs578T, and MDA-MB-468 | Multidrug resistance and apoptosis pathways | 7 |
| Mitosis | TUBB3 | Upregulated  2.65E-10 | Tongue squamous cell carcinoma HSC-3 cells | PI3K/Akt pathway | 8 |
|  | ALK | Upregulated  5.15E-05 | Non-small Cell Lung Cancer Patients | EML4-ALK fusion gene | 9 |
|  | FRY | Downregulated  0.008727047 | Not available | Not available | Not available |
|  | KNL1 | Downregulated  2.72E-15 | Breast cancer cell line MDA-MB-231, and mice xenografts models | Spindle assembly checkpoint kinase TTK | 10 |
| Receptors | PDGFR | Upregulated  0.001021801 | Human HNSCC of the oropharynx (11A) and larynx (14C) | Platelet-derived growth factor receptor α, β. Angiogenic and cytokines pathways | 11 |
|  | NOTCH1 | Upregulated  0.000739924 | Tongue squamous cell carcinoma CAL27  Pharynx squamous cell carcinoma FaDu | NOTCH pathway participates in EMT | 12 |
| Metabolism | PIK3CA | Upregulated  0.006980052 | Pancreatic cancer DU145 human cell lines | PI3K/Akt/mTOR pathway | 13 |
|  | DEPTOR | Upregulated  4.06E-10 | Esophageal squamous cell carcinoma patients, and cell lines TE-1, TE-13, EC109, KYSE510 | IRS1/PI3K/Akt/mTOR pathway | 14 |
|  | CASTOR1 | Upregulated  9.73E-09 | Not available | Not available | Not available |
|  | GSK3B | Upregulated  1.11E-05 | Human breast cancer cell lines MDA-MB-231, and MCF-7 | GSK-3β/β-Catenin Signaling Pathway | 15 |
|  | CYP24A1 | Upregulated  1.58E-07 | Pancreatic cancer patients, and HEK293T, DU145 and 22Rv1 cell lines | PI3K/AKT/mTOR pathway | 16 |
|  | IRS1 | Downregulated  5.28E-16 | Not available | Not available | Not available |
|  | SGK1 | Downregulated  5.63E-12 | Not available | Not available | Not available |
| Hypoxia | HIF1A | Upregulated  3.44E-05 | Human breast cancer patients, and cell lines MB-231 and MB-468 | HIF-1α/miR-494/Survivin signaling pathway | 17 |
| Apoptosis | NTRK1 | Upregulated  2.77E-06 | Not available | Not available | Not available |
|  | MDM2 | Upregulated  2.64E-09 | Mice-derived prostatic carcinoma cells | Apoptosis and EMT pathway | 18 |
|  | TP53 | Downregulated  5.29E-08 | Non-small cell lung cancer  A549, H460 and H1355 cells, and mice xenografts models | Apoptosis pathway | 19 |
|  | TNFRS11A | Downregulated  6.19E-07 | Lung adenocarcinoma H1299 and A549 cell lines | mTOR pathway and correlation with GPI gene expression | 20 |
|  | BCL2 | Downregulated  7.64E-11 | Prostate cancer cell lines  PC-3 | Apoptosis pathway | 21 |
| Cytokine production | STAT1 | Upregulated  2.06E-08 | Colon carcinoma cell lines HCT116, DLD1, MCF7, SKOV-3 hTERT-BJ | Interferon-related genes | 22 |
|  | IL6 | Upregulated  1.79E-05 | Prostate cancer PC3 cell line | STAT pathway | 23 |
|  | CSF2RA | Upregulated  1.07E-08 | Pharynx carcinoma-monocyte coculture FaDu–THP 1 coculture | Autophagy/IL-1β-associated pathways | 24 |
| Autophagy | CFLAR-AS1 | Upregulated  0.000536234 | Not available | Not available | Not available |
|  | ATG13 | Upregulated  6.57E-09 | Not available | Not available | Not available |
|  | ULK1 | Upregulated  4.75E-06 | Not available | Not available | Not available |
| Metastasis | SLC16A1 | Downregulated  0.005262002 | Not available | Not available | Not available |
|  | COL4A1 | Downregulated  3.28E-07 | Ovarian cancer cell line NCI/ADR-RES | MAPK–Akt, Wnt, and Notch pathways | 25 |
| Induced senescence | FGFR1 | Upregulated  1.88E-05 | Patients non-small cell lung cancer | FGFR pathway | 26 |
|  | IGF1R | Upregulated  0.008669705 | Castration-refractory prostate cancer PC3 cell line | Inflammation and/or redox-dependent pathways |  |
|  | BIRC5 | Downregulated  2.46E-14 | Cervical cancer HeLa cell line | Apoptosis pathway | 28 |

**References for Table S2**

1. Wang S, Han L, Li J, Liu Y, Wang S. Inflammatory molecules facilitate the development of docetaxel-resistant prostate cancer cells in vitro and in vivo. *Fundam Clin Pharmacol*. 2022;36(5):837-849. doi:10.1111/fcp.12773

2. Wong N, Major P, Kapoor A, et al. Amplification of MUC1 in prostate cancer metastasis and CRPC development. *Oncotarget*. 2016;7(50):83115-83133. doi:10.18632/oncotarget.13073

3. Astashchanka A, Shroka TM, Jacobsen BM. Mucin 2 (MUC2) modulates the aggressiveness of breast cancer. *Breast Cancer Res Treat*. 2019;173(2):289-299. doi:10.1007/s10549-018-4989-2

4. Fujita T, Chiwaki F, Takahashi RU, et al. Identification and characterization of CXCR4-positive gastric cancer stem cells. *PLoS One*. 2015;10(6):1-19. doi:10.1371/journal.pone.0130808

5. Sarwar M, Syed Khaja AS, Aleskandarany M, et al. The role of PIP5K1α/pAKT and targeted inhibition of growth of subtypes of breast cancer using PIP5K1α inhibitor. *Oncogene*. 2019;38(3):375-389. doi:10.1038/s41388-018-0438-2

6. Singh SK, Lillard JW, Singh R. Reversal of drug resistance by planetary ball milled (PBM) nanoparticle loaded with resveratrol and docetaxel in prostate cancer. *Cancer Lett*. 2018;427:49-62. doi:10.1016/j.canlet.2018.04.017

7. Jung H, Kim D, Kang YY, Kim H, Lee JB, Mok H. CpG incorporated DNA microparticles for elevated immune stimulation for antigen presenting cells. *RSC Adv*. 2018;8(12):6608-6615. doi:10.1039/c7ra13293j

8. Cui J, Wang H, Zhang X, Sun X, Zhang J, Ma J. Exosomal miR-200c suppresses chemoresistance of docetaxel in tongue squamous cell carcinoma by suppressing TUBB3 and PPP2R1B. *Aging (Albany NY)*. 2020;12(8):6756-6773. doi:10.18632/AGING.103036

9. Morodomi Y, Takenoyama M, Inamasu E, et al. Non-small cell lung cancer patients with EML4-ALK fusion gene are insensitive to cytotoxic chemotherapy. *Anticancer Res*. 2014;34(7):3825-3830.

10. Maia ARR, De Man J, Boon U, et al. Inhibition of the spindle assembly checkpoint kinase TTK enhances the efficacy of docetaxel in a triple-negative breast cancer model. *Ann Oncol*. 2015;26(10):2180-2192. doi:10.1093/annonc/mdv293

11. Aderhold, C., Umbreit, C., Faber, A., Sauter, A., Sommer, J.U., Birk, R., Erben, P., Hofheinz, R.D., Stern-Straeter, J., Hoermann, K. and Schultz J. Chemotherapeutic Alteration of VEGF, PDGF and PDGFRα/β Expression Under 5-FU vs. Docetaxel in HPV-transformed Squamous Cell Carcinoma Compared To HPV-negative HNSCC In Vitro. *Oncol Reports Anticancer Res*. 2013;33(5):1951-1961. doi:10.3892/or.2011.1499

12. Zhao ZL, Zhang L, Huang CF, et al. NOTCH1 inhibition enhances the efficacy of conventional chemotherapeutic agents by targeting head neck cancer stem cell. *Sci Rep*. 2016;6(March):1-12. doi:10.1038/srep24704

13. Liu Z, Zhu G, Getzenberg RH, Veltri RW. The upregulation of PI3K/Akt and MAP kinase pathways is associated with resistance of microtubule-targeting drugs in prostate cancer. *J Cell Biochem*. 2015;116(7):1341-1349. doi:10.1002/jcb.25091

14. Dong X, Wang L, Han Z, Zhou L, Shan L, Ding Y. Different functions of DEPTOR in modulating sensitivity to chemotherapy for esophageal squamous cell carcinoma. *Exp Cell Res*. 2017;353(1):35-45. doi:10.1016/j.yexcr.2017.03.003

15. Zhang X, Zhong S, Xu Y, et al. MicroRNA-3646 contributes to docetaxel resistance in human breast cancer cells by GSK-3β/β-catenin signaling pathway. *PLoS One*. 2016;11(4):1-14. doi:10.1371/journal.pone.0153194

16. Yin H, Qin H, Yang L, et al. circCYP24A1 promotes Docetaxel resistance in prostate Cancer by Upregulating ALDH1A3. *Biomark Res*. 2022;10(1):1-20. doi:10.1186/s40364-022-00393-1

17. Li H, Su X, Li J, et al. Hypoxia induces docetaxel resistance in triple-negative breast cancer via the HIF-1α/miR-494/Survivin signaling pathway. *Neoplasia (United States)*. 2022;32(C):100821. doi:10.1016/j.neo.2022.100821

18. Slabáková E, Kharaishvili G, Smejová M, et al. Opposite regulation of MDM2 and MDMX expression in acquisition of mesenchymal phenotype in benign and cancer cells. *Oncotarget*. 2015;6(34):36156-36171. doi:10.18632/oncotarget.5392

19. Kuo WT, Tu DG, Chiu LY, Sheu GT, Wu MF. High pemetrexed sensitivity of docetaxel-resistant A549 cells is mediated by TP53 status and downregulated thymidylate synthase. *Oncol Rep*. 2017;38(5):2787-2795. doi:10.3892/or.2017.5951

20. Yang Z, Zhu J, Yang T, et al. Comprehensive analysis of the lncRNAs-related immune gene signatures and their correlation with immunotherapy in lung adenocarcinoma. *Br J Cancer*. 2023;129(9):1397-1408. doi:10.1038/s41416-023-02379-8

21. Cao W, Shiverick KT, Namiki K, et al. Docetaxel and bortezomib downregulate Bcl-2 and sensitize PC-3-Bcl-2 expressing prostate cancer cells to irradiation. *World J Urol*. 2008;26(5):509-516. doi:10.1007/s00345-008-0289-5

22. Kolosenko I, Fryknäs M, Forsberg S, et al. Cell crowding induces interferon regulatory factor 9, which confers resistance to chemotherapeutic drugs. *Int J Cancer*. 2015;136(4):E51-E61. doi:10.1002/ijc.29161

23. Mahon KL, Lin HM, Castillo L, et al. Cytokine profiling of docetaxel-resistant castration-resistant prostate cancer. *Br J Cancer*. 2015;112(8):1340-1348. doi:10.1038/bjc.2015.74

24. Hsieh CY, Lin CC, Huang YW, et al. Macrophage secretory IL-1β promotes docetaxel resistance in head and neck squamous carcinoma via SOD2/CATICAM1 signaling. *JCI Insight*. 2022;7(23):1-19. doi:10.1172/jci.insight.157285

25. Vert A, Castro J, Ribó M, Vilanova M, Benito A. Transcriptional profiling of NCI/ADR-RES cells unveils a complex network of signaling pathways and molecular mechanisms of drug resistance. *Onco Targets Ther*. 2018;11:221-237. doi:10.2147/OTT.S154378

26. Morgensztern D, Karaseva N, Felip E, Delgado I, Burdaeva O, Dómine M, Lara P, Paik PK, Lassen U, Orlov S, Trigo J. An open-label phase IB study to evaluate GSK3052230 in combination with paclitaxel and carboplatin, or docetaxel, in FGFR1-amplified non-small cell lung cancer. Lung Cancer. 2019 Oct 1;136:74-9

27. Zhang D, Cui Y, Niu L, Xu X, Tian K, Young CY, Lou H, Yuan H. Regulation of SOD2 and β-arrestin1 by interleukin-6 contributes to the increase of IGF-1R expression in docetaxel resistant prostate cancer cells. European Journal of Cell Biology. 2014 Jul 1;93(7):289-98.

28. Han TL, Sha H, Ji J, Li YT, Wu DS, Lin H, Hu B, Jiang ZX. Depletion of Survivin suppresses docetaxel-induced apoptosis in HeLa cells by facilitating mitotic slippage. *Scientific Reports*. 2021 Jan 27;11(1):2283.


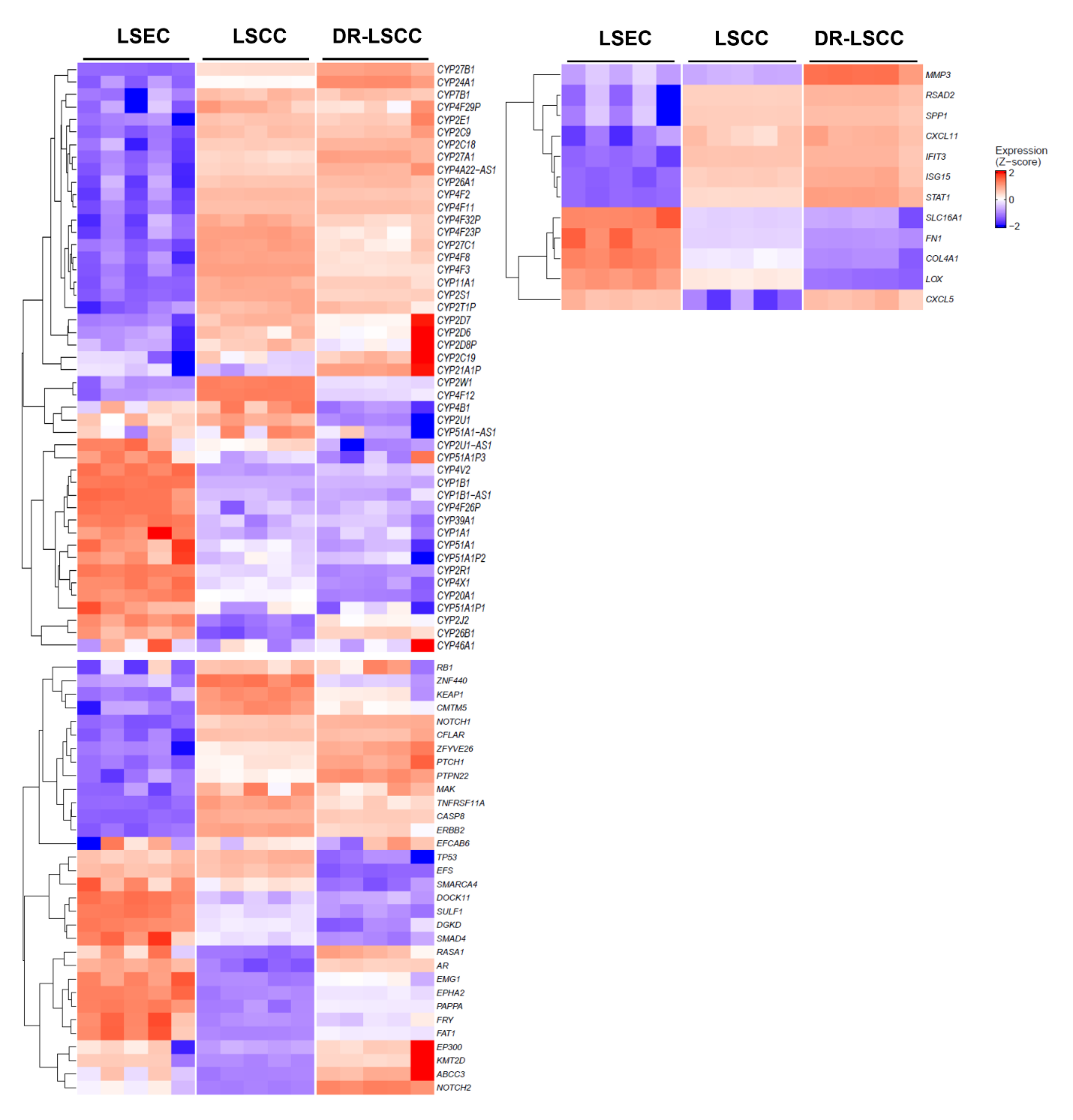


Figure S1. Heatmap of specific differences on drug metabolism (CYP family), HNC biomarkers, oncogenes, and tumor suppressors wit Z-score (-2 to 2). Red = upregulation, Blue = downregulation. A linear model was used to obtain differentially expressed genes. A linear model was used to obtain differentially expressed genes. For all genes and pathways, significance is defined as *p*-adjusted <0.05.


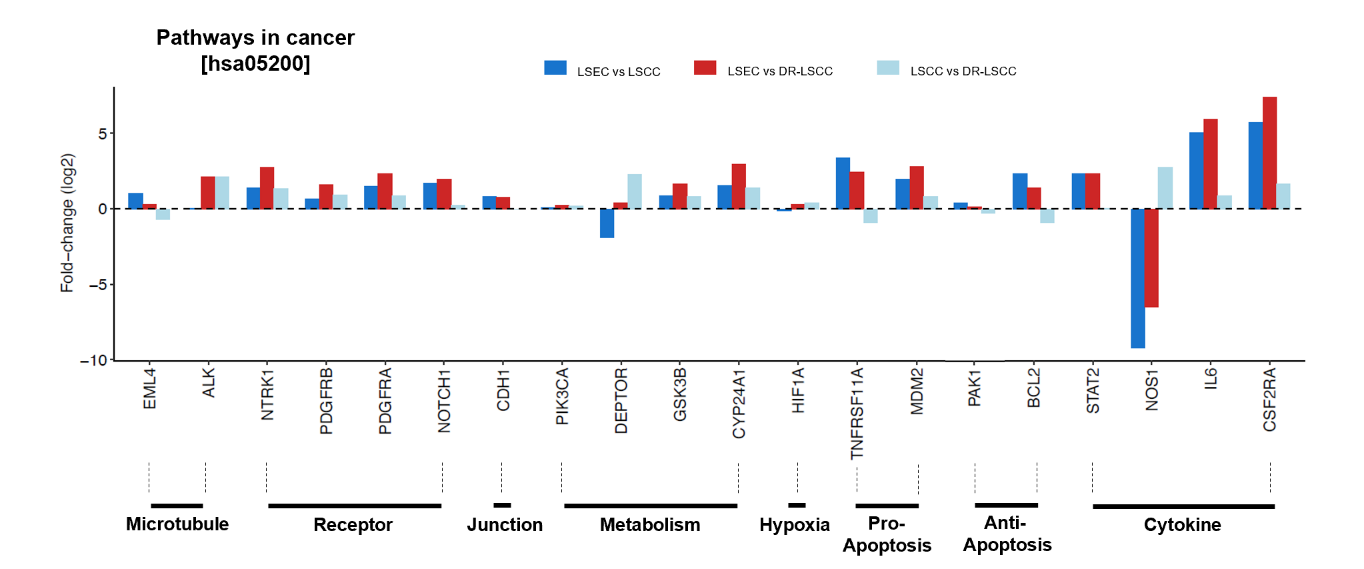


Figure S2. Genes of interest from cancer pathway [hsa05200]. Blue = LSEC vs. LSCC; Red = LSEC vs. DR-LSCC; Light blue = LSCC vs DR-LSCC. Bars represent fold changes in gene expression (log 2). A linear model was used to obtain differentially expressed genes. For all genes and pathways, significance is defined as *p*-adjusted <0.05.


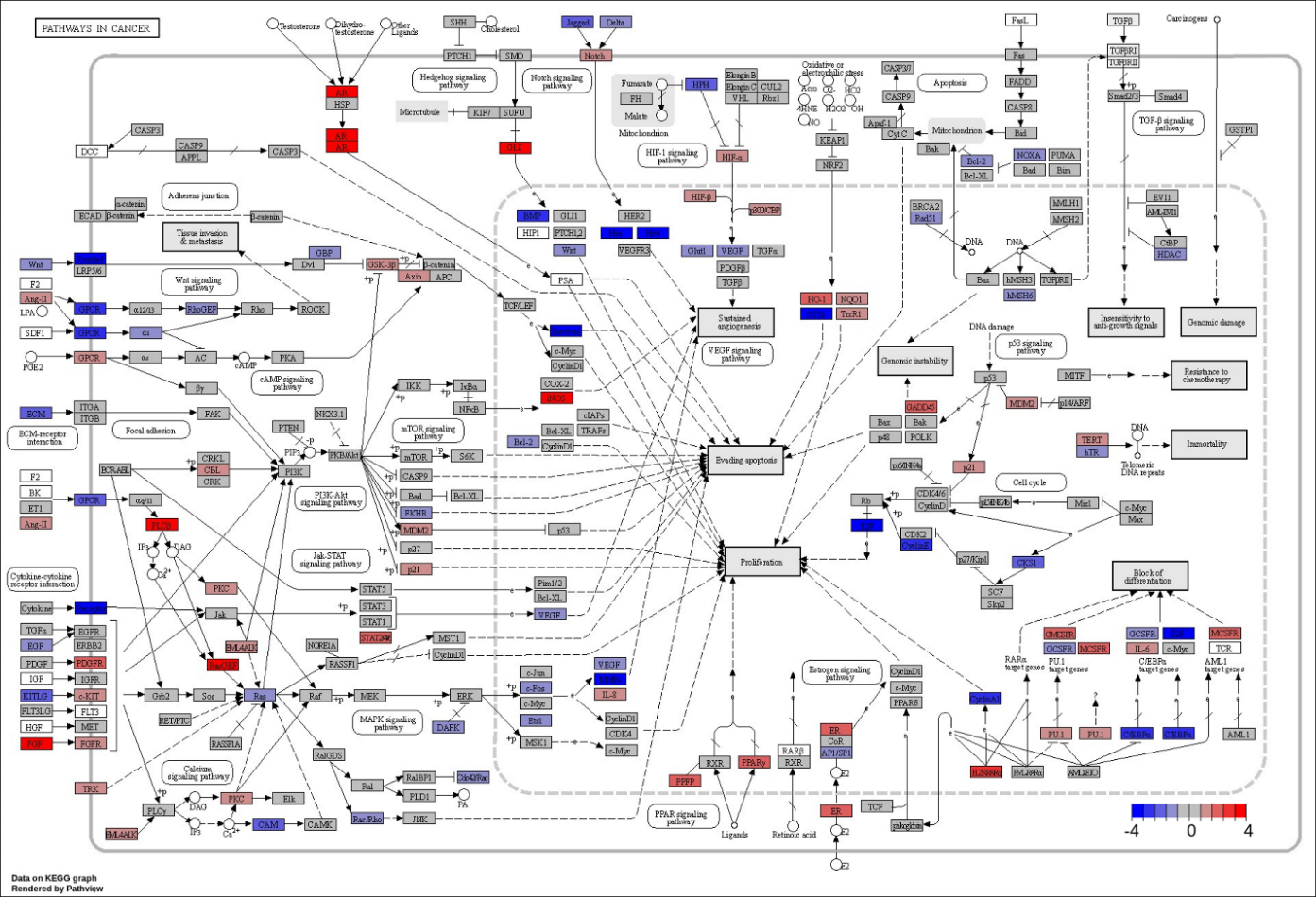


Figure S3. Cancer pathway [hsa05200] LSCC vs DR-LSCC. Red = upregulation, Blue = downregulation. Color represents fold changes in gene expression. A linear model was used to obtain differentially expressed genes. For all genes and pathways, significance is defined as *p*-adjusted <0.05.


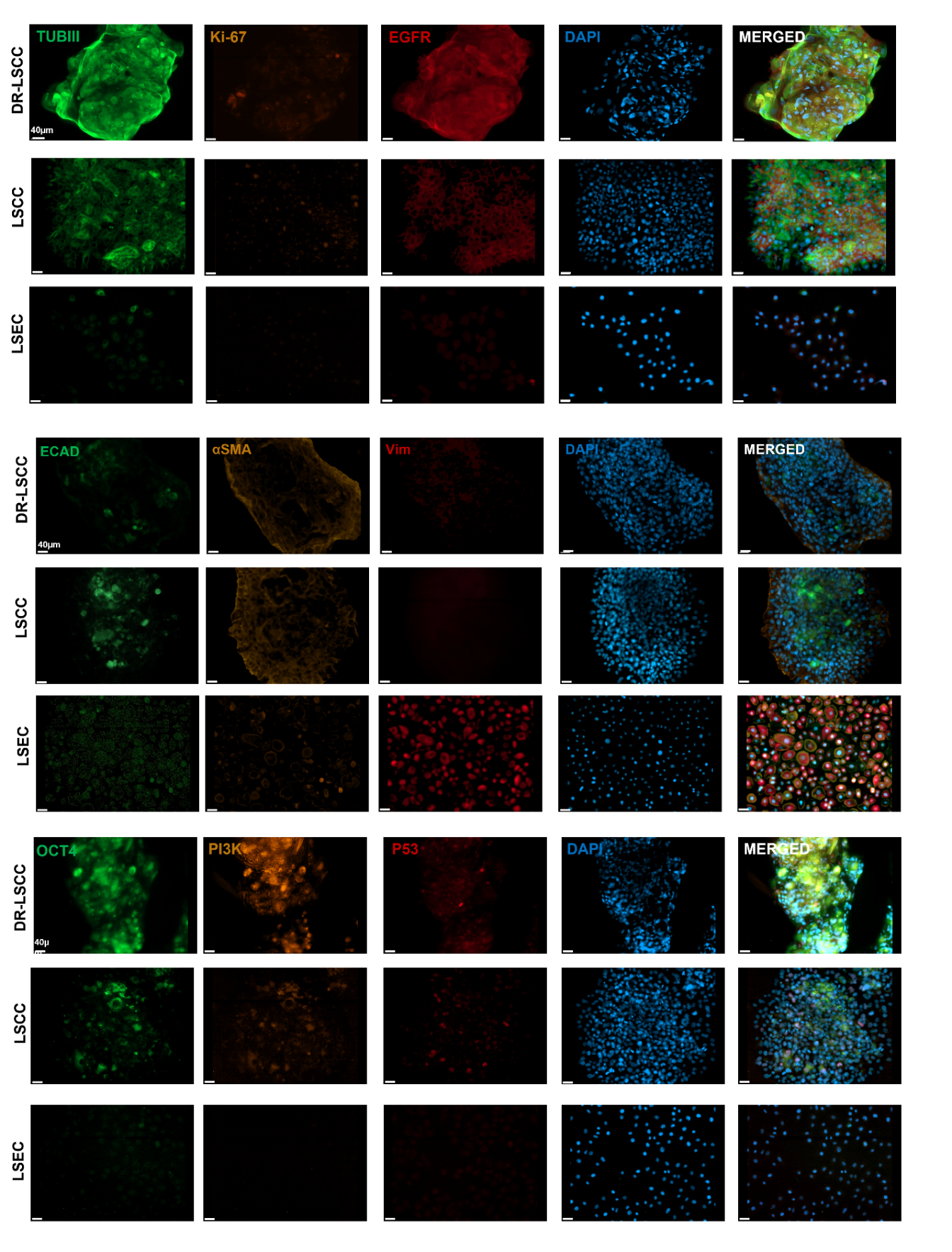


Figure S4. Chemoresistance marker expressions of LSEC, LSCC, and DR-LSCC was carried out via the staining of βIII-tubulin (referred as TUBIII), ki-67, EGFR, vimentin, E-Cadherin, α-Smooth Muscle Actin, PI3KCA, P53, and Oct-4. Scale bar = 40 µm.

**
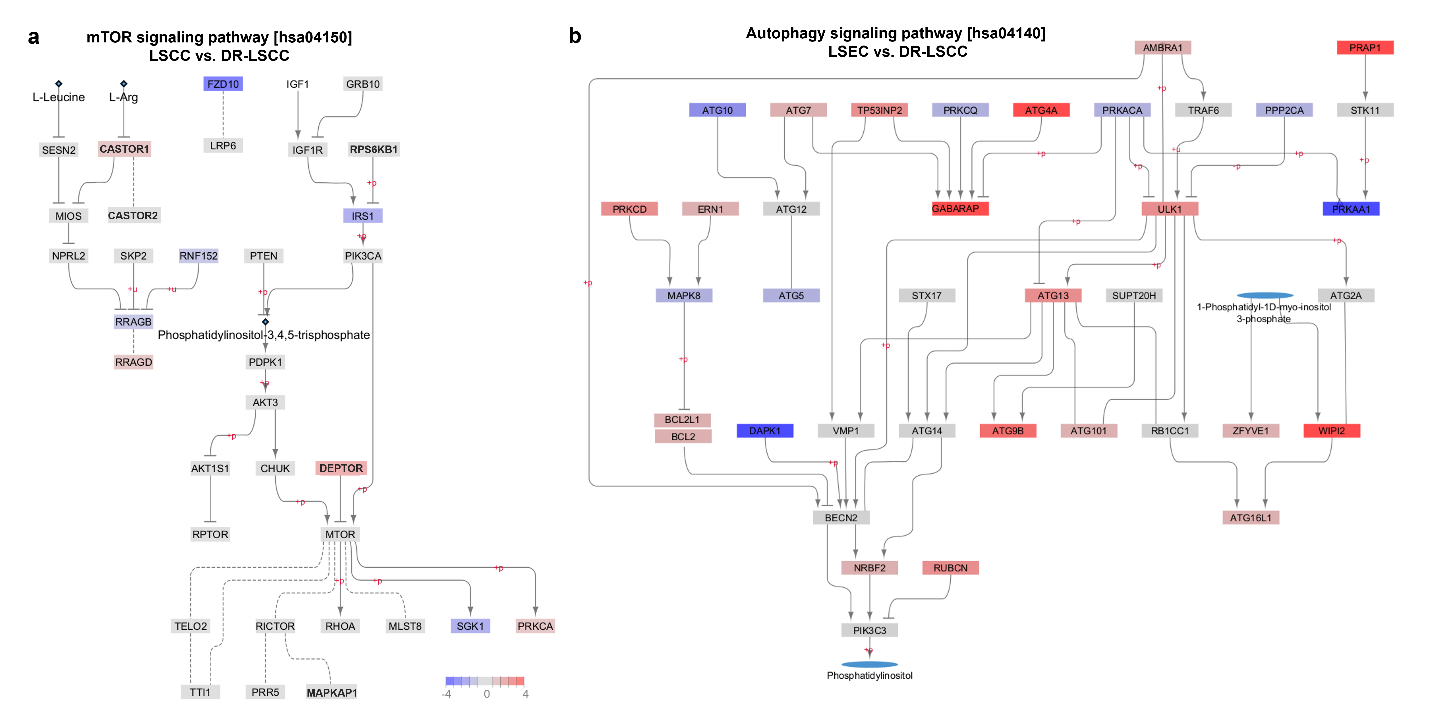
**

**Figure S5.** Simplified KEGG signaling pathways. (a) Representative LSCC vs. DR-LSCC mTOR pathway [hsa04150], and LSEC vs. DR-LSCC autophagy pathway [hsa4140]. Color bar of fold-change (-4 to 4). Red = upregulation; Blue = downregulation. A linear model was used to obtain differentially expressed genes**.** For all genes and pathways, significance is defined as *p*-adjusted <0.05**.**


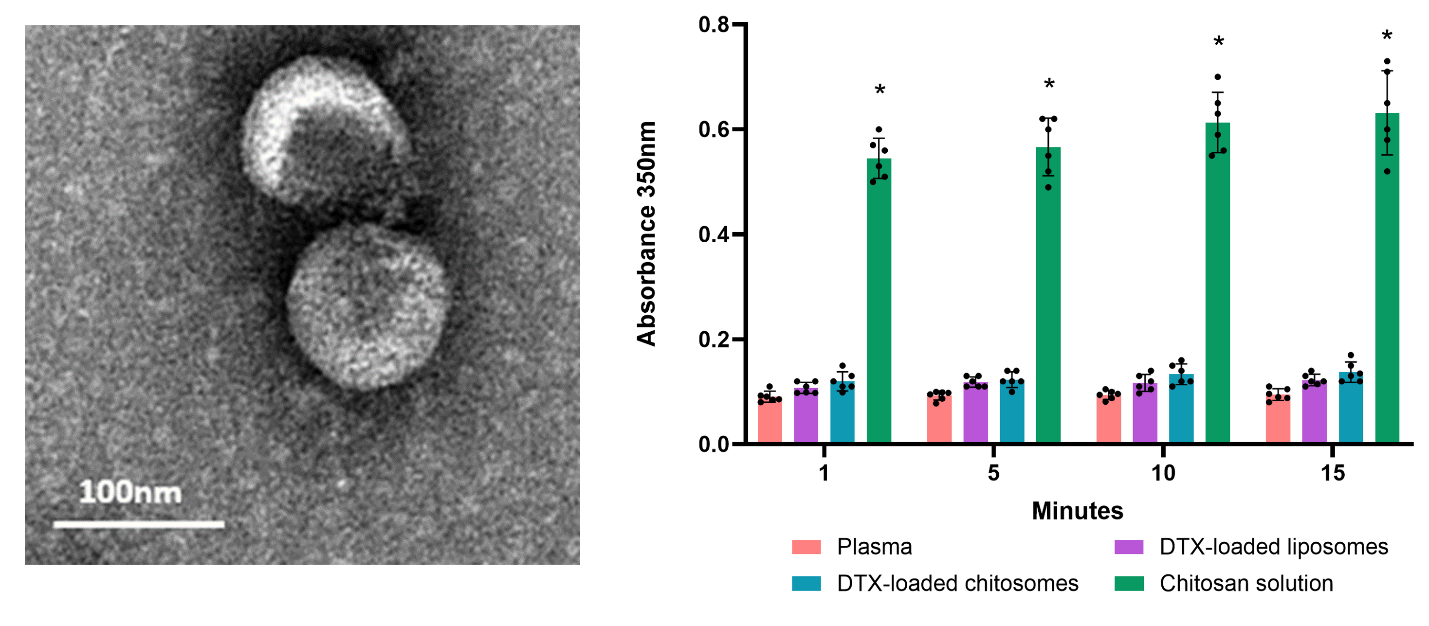


Figure S6. Transmission electron images of the DTX-loaded chitosomes along with ATPP coagulation assay. DTX-loaded chitosomes showed absence of coagulation effect in comparison to 6mg/mL chitosan solution. Analyzed groups comprised APTT reagent added to (1) human plasma, (2) human plasma/DTX-loaded liposomes, (3) human plasma/DTX-loaded chitosomes, and (4) human plasma/chitosan solution. Two-way ANOVA, Bonferroni’s multiple comparisons test as post-hoc test (n = 6, * *p* < 0.05).

**
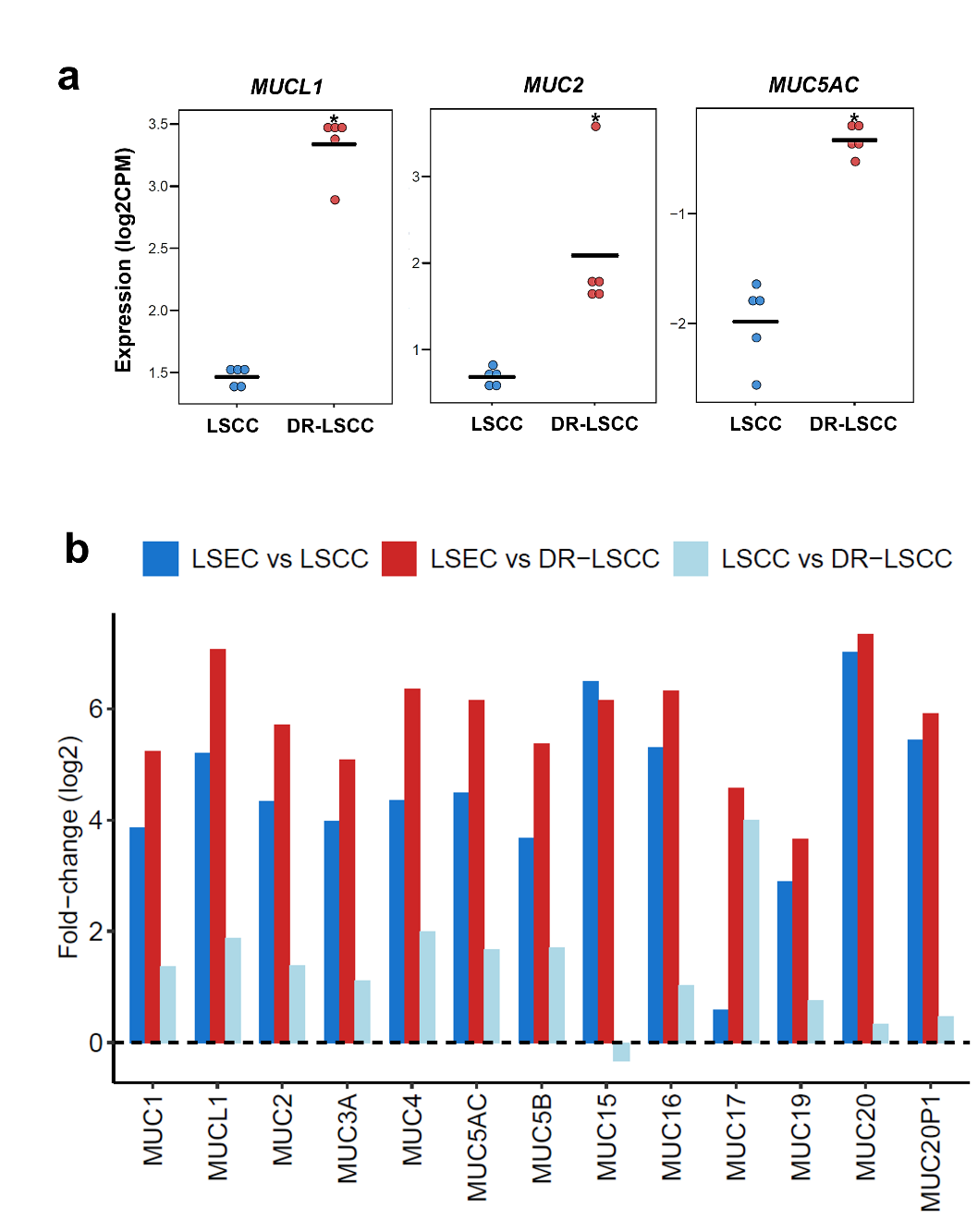
**


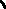


**Figure S7.** Mucin-related genes. (a) Representative upregulated mucin-associated genes LSCC vs. DR-LSCC. Gene set enrichment analysis (GSEA) based on pre-ranked gene list by t-statistic (n = 5, * *p* < 0.05**)**. (b) Genes of interest from the mucin pathway. Blue = LSEC vs. LSCC; Red = LSEC vs. DR-LSCC; Light blue = LSCC vs DR-LSCC. Bars represent fold changes in gene expression (log 2). A linear model was used to obtain differentially expressed genes**.** For all genes and pathways, significance is defined as *p*-adjusted <0.05**.**


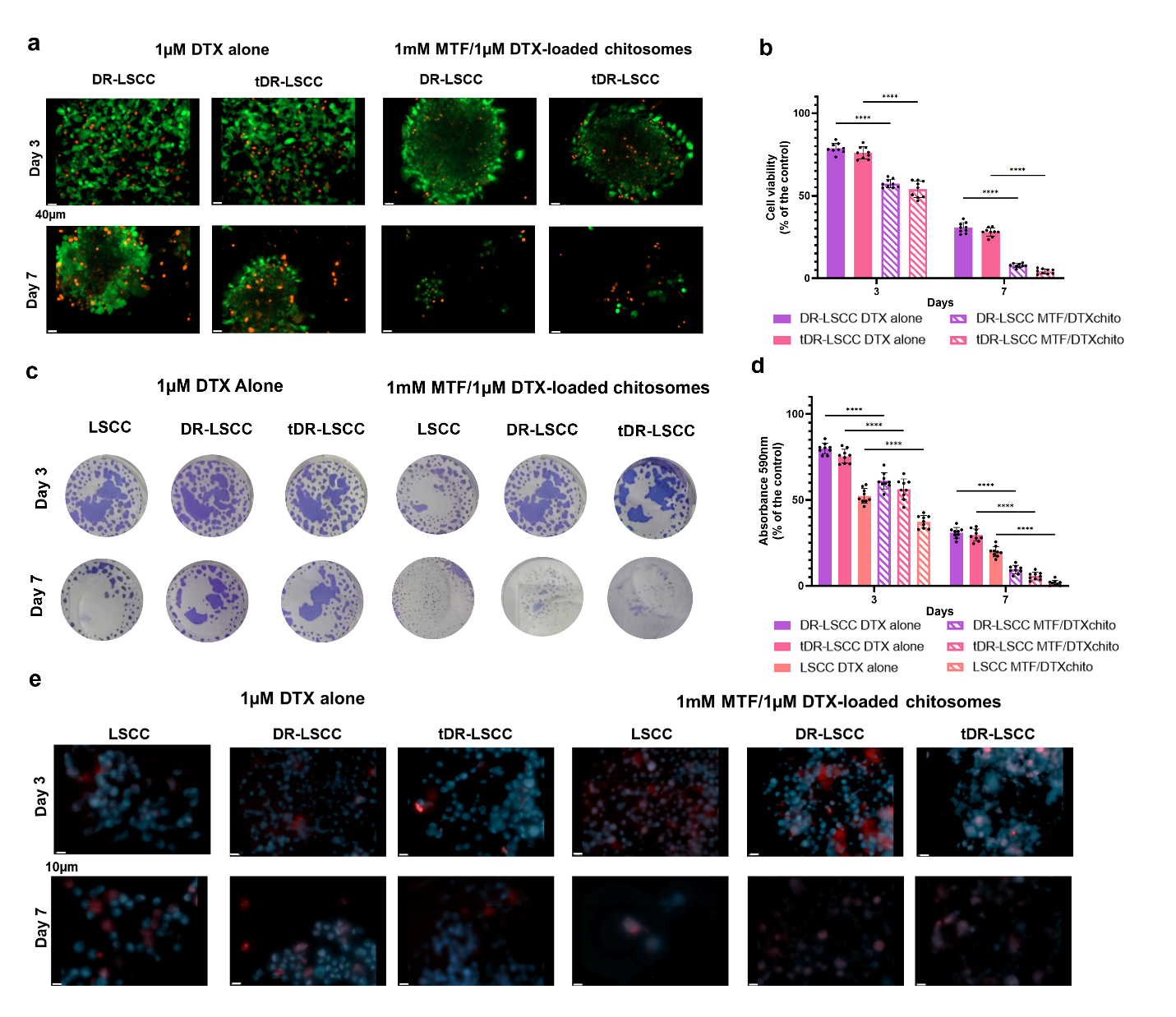
 Figure S8. Combined metformin/docetaxel treatment with resistant laryngeal cancer cells after a single freeze/thaw cycle. Cytotoxic evaluation via (a) LIVE/DEAD staining. Green = live cells; Orange = dead cells. Scale bar = 40µm (b) MTT cytotoxic analysis. DR-LSCC data from Figure 8c was included for cell viability comparisons. (c-d) Clonogenic studies, and (e) Autophagy studies on laryngeal cancer cells with scale bar = 10µm. Blue = autophagy activity; Red = dead cells. Two-way ANOVA, Bonferroni’s multiple comparisons test as post-hoc test (n = 9, **** *p* < 0.0001). tDR-LSCC = thawed DR-LSCC.


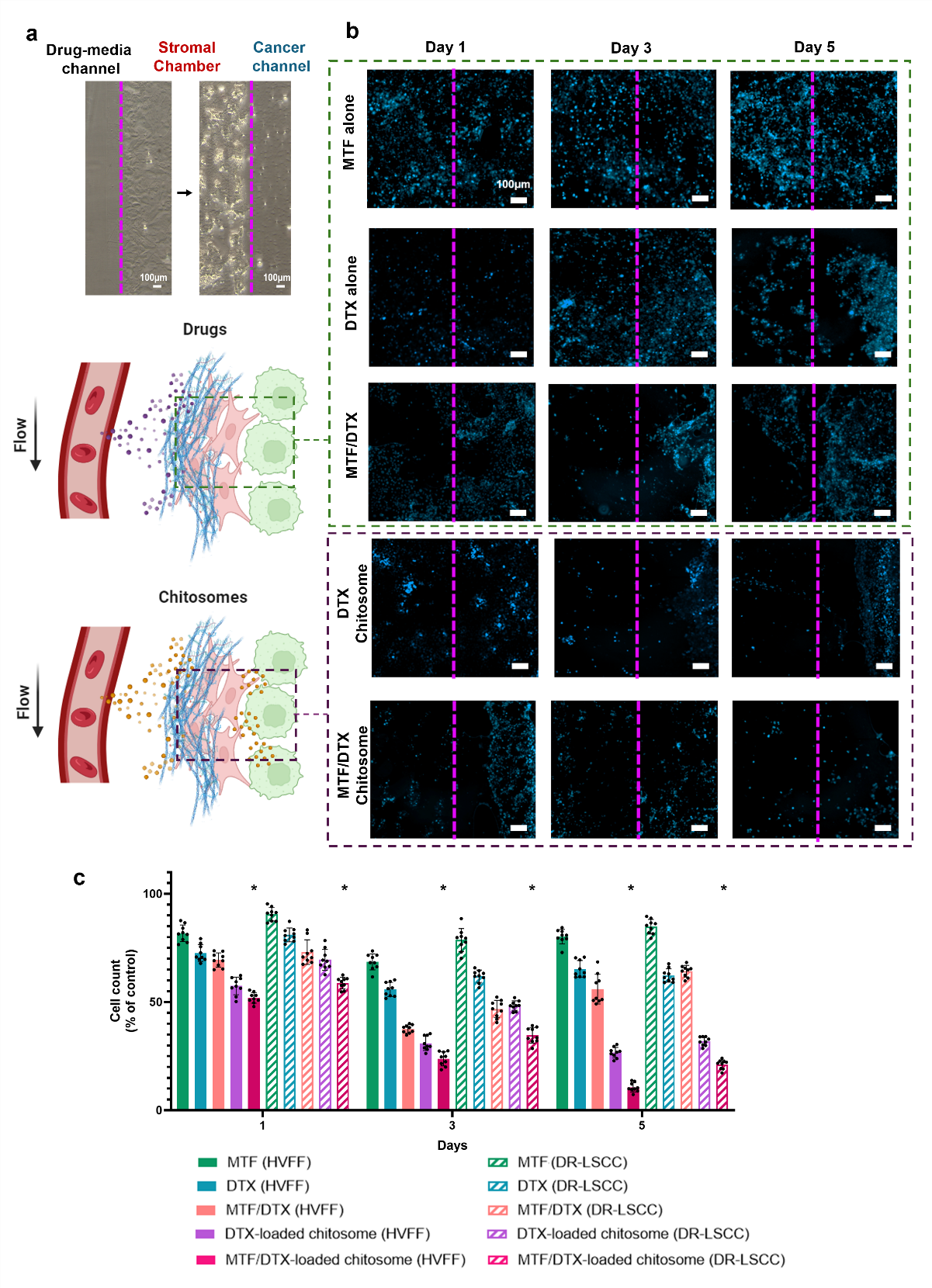


Figure S9. Cytotoxic effect of the combination MTF/DTX therapy of the tumor-on-a-chip. (a) Brightfield images of the microfluidic device and schematic representation of the drug uptake. Cytotoxic effect via (b) DAPI inspection up to 5 days. Scale bar = 100 µm. Magenta line = collagen gel limit. (c) Cell count using Spot detection algorithm on DAPI images up to 5-day inspection. Two-way ANOVA, Bonferroni’s multiple comparisons test as post-hoc test (n = 9, * *p* < 0.05, compared to respective cell controls with combination therapy).


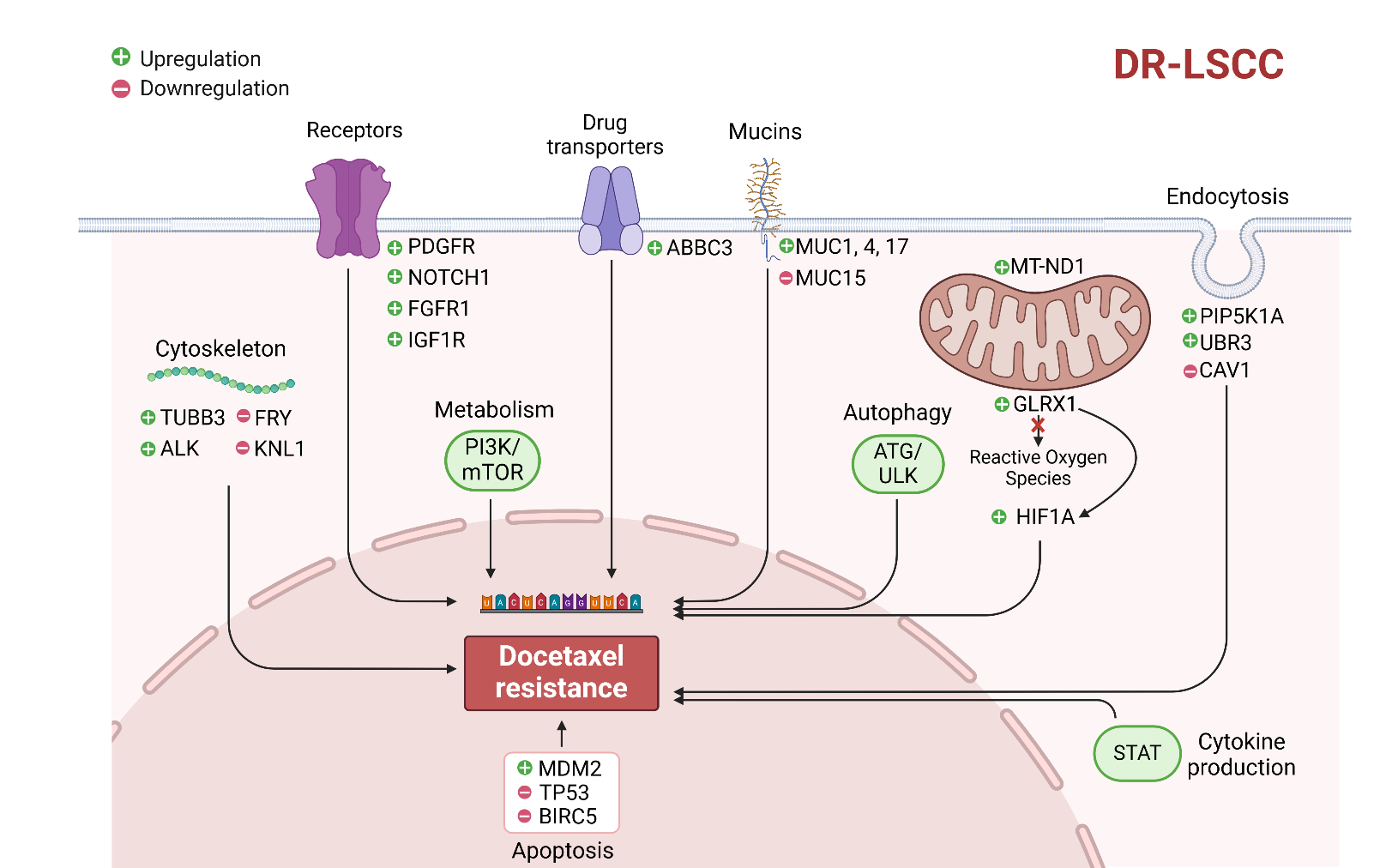


Figure S10. Representative genes associated with docetaxel resistance in DR-LSCC. Chemotherapy-induced senescence: FGFR1, IGF1R, and BIRC5. Figure created with BioRender.com.


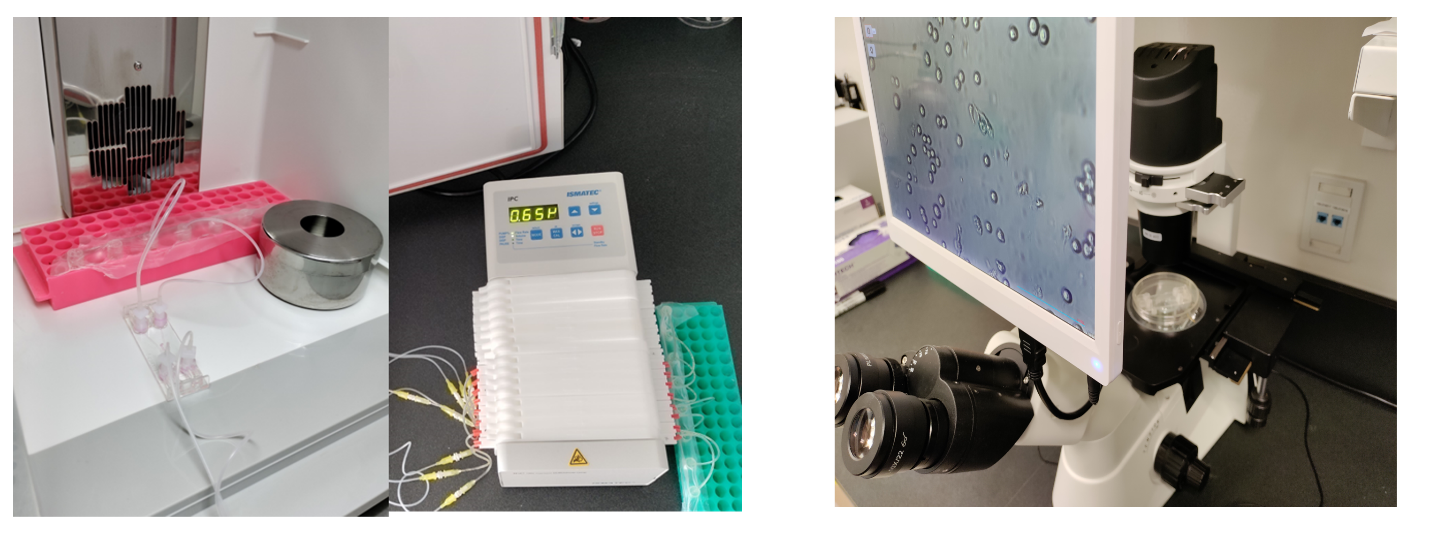


Figure S11. Microfluidic setup of the cancer/stromal co-culture and cell seeding evaluation.

Video S1. Supplementary video of microfluidic setup and injection of fluorescent chitosomes. Provisional link prior publication: [Video S1.mp4](https://mcgill-my.sharepoint.com/:v:/g/personal/christian_moyagarcia_mail_mcgill_ca/EYymoI9NahdArf1dWa7C5yEBNch34PK-vaY1iBeIucssLw?e=QYJnjt)
